# External validation of nomograms including PSMA PET information for the prediction of lymph node involvement of prostate cancer

**DOI:** 10.1007/s00259-025-07241-y

**Published:** 2025-04-02

**Authors:** Tessa D. Van Bergen, Arthur J. A. T. Braat, Rick Hermsen, Joris G. Heetman, Lieke Wever, Jules Lavalaye, Maarten Vinken, Clinton D. Bahler, Mark Tann, Claudia Kesch, Tugce Telli, Peter Ka-Fung Chiu, Kwan Kit Wu, Fabio Zattoni, Laura Evangelista, Francesco Ceci, Marcin Miszczyk, Pawel Rajwa, Francesco Barletta, Giorgio Gandaglia, Jean-Paul A. Van Basten, Matthijs J. Scheltema, Harm H. E. Van Melick, Roderick C. N. Van den Bergh, Cornelis A. T. Van den Berg, Giancarlo Marra, Timo F. W. Soeterik, on behalf of the EAU-YAU Prostate Cancer Working Party

**Affiliations:** 1 Computational Imaging Group for MR Diagnostics and Therapy, Center for Image Sciences, University Medical Center Utrecht, Utrecht, The Netherlands; 2 Department of Nuclear Medicine and Radiology, Division of Imaging and Oncology, University Medical Center, Utrecht, The Netherlands; 3 Department of Nuclear Medicine, Canisius Wilhelmina Hospital, Nijmegen, The Netherlands; 4 Department of Urology, St. Antonius Hospital, Nieuwegein, Utrecht, The Netherlands; 5 Department of Nuclear Medicine, St. Antonius Hospital, Nieuwegein, Utrecht, The Netherlands; 6 Department of Urology, Indiana University Medical Center, Indianapolis, USA; 7 Department of Radiology and Imaging Sciences, Indiana University Medical Center, Indianapolis, USA; 8 Department of Urology, University Hospital Essen, Essen German Cancer Consortium (DKTK), Essen, Germany; 9 Department of Nuclear Medicine, University Hospital Essen, Essen, Germany; 10 West German Cancer Center (WTZ), German Cancer Consortium (DKTK), Essen, Germany; 11 S. H. Ho Urology Centre, Department of Surgery, The Chinese University of Hong Kong, Hong Kong, China; 12 Department of Nuclear Medicine and PET, Hong Kong Sanatorium and Hospital, Hong Kong, China; 13 Department of Surgery, Oncology, and Gastroenterology, Urological Unit, University of Padova, Padova, Italy; 14 Department of Biomedical Sciences, Humanitas University, Pieve Emanuele, Milan, Italy; 15 Division of Nuclear Medicine, IRCCS Humanitas Research Hospital, Milan, Italy; 16 Division of Nuclear Medicine and Theranostics, IEO European Institute of Oncology, IRCCS, Milan, Italy; 17 Department of Urology, Comprehensive Cancer Center, Medical University of Vienna, Vienna, Austria; 18 Collegium Medicum - Faculty of Medicine, WSB University, Dąbrowa Górnicza, Poland; 19 Second Department of Urology, Centre of Postgraduate Medical Education, Warsaw, Poland; 20 Division of Surgery & Interventional Science, University College London, London, UK; 21 Division of Oncology/Unit of Urology, Soldera Prostate Cancer Lab, URI, IRCCS San Raffaele Scientific Institute, Vita-Salute San Raffaele University, Milan, Italy; 22 Department of Urology, Canisius Wilhelmina Hospital, Nijmegen, The Netherlands; 23 Department of Urology, Erasmus Medical Center, Rotterdam, The Netherlands; 24 Department of Urology, University Hospital S Giovanni Battista, Azienda Ospedaliero Universitaria Città della Salute e della Scienza di Torino, Turin, Italy; 25 Department of Radiation Oncology, University Medical Center Utrecht, Utrecht, The Netherlands

**Keywords:** PSMA PET/CT, Prostate cancer, Nomogram, Lymph node involvement, External validation

## Abstract

**Background:**

Novel nomograms predicting lymph node involvement (LNI) of prostate cancer (PCa) including PSMA PET information have been developed. However, their predictive accuracy in external populations is still unclear.

**Purpose:**

To externally validate four LNI nomograms including PSMA PET parameters (three Muehlematter models and the Amsterdam-Brisbane-Sydney model) as well as the Briganti 2012 and MSKCC nomograms.

**Methods:**

Patients with histologically confirmed PCa undergoing preoperative MRI and PSMA PET/CT before radical prostatectomy (RP) and extended pelvic lymph node dissection (ePLND) were included. Model discrimination (AUC), calibration and net benefit using decision curve analysis were determined for each nomogram.

**Results:**

A total of 437 patients were included, comprising 0.7% with low-risk disease, 39.8% with intermediate-risk disease, and 59.5% with high-risk disease. Among them, 86 out of 437 (19.7%) had pN1 disease. The sensitivity and specificity of PSMA PET/CT for the detection of LNI were 47.7% (95% CI: 36.8–58.7) and 95.4% (95% CI: 92.7–97.4), respectively. Among predictive models, the Amsterdam-Brisbane-Sydney model achieved the highest discrimination (AUC: 0.81, 95% CI: 0.76–0.86), followed by Muehlematter Model 1 (AUC: 0.79, 95% CI: 0.74–0.85), both with good calibration but slight systematic overestimation of risks across all thresholds. The MSKCC and Briganti 2012 models had AUCs of 0.68 (95% CI: 0.61–0.74) and 0.67 (95% CI: 0.61–0.73), respectively, and both had moderate calibration. Decision curve analysis indicated that the Amsterdam-Brisbane-Sydney model provided superior net benefit across thresholds of 5–20%, followed by the Muehlematter Model 1 nomogram showing benefit in the 14–20% range. Using thresholds of 8% for the Amsterdam-Brisbane-Sydney nomogram and 15% for Muehlematter Model 1, ePLND could be spared in 15% and 16% of patients, respectively, without missing any LNI cases.

**Conclusion:**

External validation of the Muehlematter Model 1 and Amsterdam-Brisbane-Sydney nomograms for predicting LNI confirmed their strong model discrimination, moderate calibration, and good clinical utility, supporting their reliability as tools to guide clinical decision-making.

**Supplementary Information:**

The online version contains supplementary material available at 10.1007/s00259-025-07241-y.

## Introduction

Accurate nodal staging of prostate cancer (PCa) is essential for determining disease prognosis and guiding clinical decisions in patients considered for treatment with curative intent [1]. While the advent of prostate-specific membrane antigen positron emission tomography (PSMA PET) has significantly enhanced the detection of pelvic lymph node involvement (LNI), its sensitivity remains suboptimal, ranging from 40 to 50% [2–5]. Consequently, extended pelvic lymph node dissection (ePLND) continues to be the most accurate method for nodal staging.

However, ePLND is associated with risks of surgical complications, including symptomatic lymphocele formation (in up to 18%), bleeding (2.7%), infections (3.6%), and ureteric nerve injury (0.8%) [6, 7]. Additionally, ePLND has been identified as an independent risk factor for major complications in surgically treated PCa patients [8]. The therapeutic benefit of ePLND is also under debate, as a definitive improvement in oncologic outcomes is yet to be confirmed in randomised controlled trials [9–11]. Therefore, careful patient selection is crucial, balancing the potential risks of ePLND against its prognostic value and any therapeutic advantages that might arise from removing positive nodes.

Numerous nomograms for predicting LNI have been developed, with the MSKCC and Briganti 2012 models being among the most widely validated and consistently demonstrating favorable performance [12–15]. The more recent Briganti 2019 nomogram, which incorporates prebiopsy MRI and targeted biopsy core data, has also shown promising performance in external populations [16–18]. Integrating PSMA PET information with clinical and (targeted) biopsy data holds the potential to further enhance LNI prediction in contemporary PCa cohorts [19, 20]. Recently, two novel nomograms incorporating PSMA PET findings have been developed. However, their performance in external populations has not yet been thoroughly evaluated [21, 22].

This study aims to externally validate and compare six different nomograms, including those incorporating PSMA PET data, using a large, international, and multi-institutional patient cohort not involved with the development of the original nomograms to predict lymph node metastatic (pN1) disease in patients undergoing ePLND. The study will also evaluate the inter-tracer variability in model performance by assessing the models incorporating PSMA PET/CT findings for [^68^Ga]Ga-PSMA-11 and [^18^F]F-PSMA-1007 separately.

## Methods

### Patient population

Data collection was initiated after the study was approved by the local institutional review board. Patients with histologically proven PCa treated with robot-assisted radical prostatectomy (RARP) with ePLND, undergoing preoperative PSMA PET and MRI, from 2016 to 2022 at seven tertiary referral centers worldwide were included. Patients who had prior treatment for PCa (e.g. systemic, radiation, or focal therapy) were excluded.

### MRI procedures and interpretation

Radiological reporting was conducted by uro-radiologists specialised in prostate MRI reading according to the PI-RADS version 2.1 guidelines [23]. Both multiparametric MRI (mpMRI), which includes anatomical T1-weighted (T1W) and T2-weighted (T2W) sequences along with functional sequences such as diffusion-weighted imaging (DWI), apparent diffusion coefficient (ADC) mapping, and dynamic contrast-enhanced imaging (DCE), and biparametric MRI (bpMRI), which comprises DWI and T2W sequences without DCE, were allowed. Radiologists assessed the local tumour stage and documented the presence of extraprostatic extension (EPE) and seminal vesicle invasion (SVI).

### PSMA PET/CT protocols and procedures

The PSMA PET scans were performed at the tertiary referral centers according to the local previously described protocols [24]. The PET scans were made from mid-thigh to skull base and were made in combination with a low-dose CT scan or a contrast-enhanced CT scan for anatomic correlation. The evaluation of all PSMA PET scans at each referral center was performed by an experienced nuclear medicine physician (> 5-year experience and/or > 500 studies). The radioligands used were [^68^Ga]Ga-PSMA-11 and [^18^F]F-PSMA-1007. Images were acquired according to the European Association of Nuclear Medicine/Society of Nuclear Medicine and Molecular Imaging guideline [25].

### Radical prostatectomy and extended pelvic lymph node dissection

All patients underwent RARP and ePLND. The ePLND template included removal of nodes overlying the external iliac vessels, internal iliac artery, and the nodes located within the obturator fossa [26]. At the discretion of the local surgical teams, template modification (e.g. expansion to super-extended PLND) was allowed [8].

### Histopathological evaluation

Histopathological processing was performed following EAU-ISUP prostate cancer guidelines, using either conventional or whole-mount sections based on local protocol [1]. Histopathological evaluation and grading were done by local uropathologists according to the ISUP guidelines [27]. All resected nodal tissue was submitted for evaluation, which was performed by experienced uro-pathologists. The total number of lymph nodes found in the tissue and the number of nodes containing prostate cancer metastasis were assessed. The histopathological evaluation of lymph node specimens was performed in accordance with the ISUP consensus [28].

### Covariates and endpoints

Preoperative serum PSA values, clinical stage assessed by digital rectal examination (DRE) by the attending urologist and MRI stage were collected. Total number of systematic and/or target biopsies taken, and relative number of positive biopsy cores were collected on whole gland and side-specific level. Highest biopsy GG per prostate side was recorded. Total number of removed nodes, positive nodes and pN status were documented. The predicted outcome was histopathologically confirmed LNI (pN1). The PSMA-positive volume (PSMA_vol_, cm³) in the prostate was evaluated following the methodology described by Ferraro et al., incorporating an SUV ≥ 4 cutoff [19]. PET nodal staging was done according to the PROMISE v2 framework (miN0/miN1/miN2) [29]. Patients with suspicious nodes on PET that remained in situ after ePLND (confirmed by PSA persistence and persistent presence of the suspicious node on postoperative PSMA PET) were considered pN1 per analysis. LNI probabilities were calculated on patient level for the three Muehlematter nomograms, Amsterdam-Brisbane-Sydney, Briganti 2012 and MSKCC nomograms [14, 15, 21, 22] are provided in Table 1. The probabilities predicting the presence of LNI were calculated for the overall patient cohort, tracer subgroups and the subgroup of patients staged miN0, using each predictive model.

**Table 1:** The different models for predicting lymph node invasion at radical prostatectomy in patients with prostate cancer and the corresponding variables included

Model	Covariates
Amsterdam-Brisbane-Sydney	• Preoperative prostate-specific antigen • Highest Grade Group from systematic biopsies • Highest Grade Group from targeted biopsies • Radiological tumour stage on MRI • Percentage of systemic biopsy cores with GG ≥ 2 • Lymph node status on PSMA PET/CT
Briganti 2012	• Preoperative prostate-specific antigen • Primary biopsy Gleason grade • Secondary biopsy Gleason grade • Clinical tumour stage • Percentage of positive cores
Memorial Sloan Kettering Cancer Center	• Preoperative prostate-specific antigen • Primary biopsy Gleason grade • Secondary biopsy Gleason grade • Clinical tumour stage • Total number of positive cores • Total number of negative cores
Muehlematter Model 1 (in original paper: Model_Clinical_PET/Report)	• Preoperative prostate-specific antigen • Highest Grade Group • Prostate-specific membrane antigen positive volume • Lymph node status on PSMA PET/CT • Here the input variables are split and processed in two logistic regression models. For the ensemble model the models were combined using averaged weights optimized using nonlinear optimization
Muehlematter Model 2 (in original paper: Model_Clinical_PET_Report)	• Preoperative prostate-specific antigen • Highest Grade Group • Prostate-specific membrane antigen positive volume • Lymph node status on PSMA PET/CT
Muehlematter Model 3 (in original paper: Model_Clinical_PET)	• Preoperative prostate-specific antigen • Highest Grade Group • Prostate-specific membrane antigen positive volume

### Statistical analysis

Medians and proportions were reported for continuous and categorical variables, respectively. Model discrimination was quantified using the area under the curve (AUC) of the receiver operating characteristic (ROC). Sensitivity, specificity, positive predictive value (PPV) and negative predictive value (NPV) of PSMA PET for the detection of LNI on a patient base were determined. Calibration was assessed using calibration plots. Net benefit was determined by decision curve analysis (DCA). Rates of patients with detected and missed positive lymph nodes were established for clinically relevant thresholds (from 0 to 30%). A *p* value of < 0.05 was considered statistically significant. Missing data were handled using complete case analysis. All statistical analyses were performed using R v4.0.5 and v4.2.0 (R Project for Statistical Computing, www.r-project.org).

## Results

### Patient baseline characteristics

A total of 437 patients that met the inclusion criteria were analysed. The median age was 66 years (IQR: 62–71), and the median PSA level was 9.9 ng/mL (IQR: 6.7–17). The cohort consisted of 0.7% low-risk, 39.8% intermediate-risk, and 59.5% high-risk patients according to the European Association of Urology (EAU) risk groups, respectively. Radioligand [^68^Ga]Ga-PSMA-11 was used in 59% of patients and [^18^F]F-PSMA-1007 in 41% of the patients (Table 2). Overall, the median number of lymph nodes removed during ePLND was 17 (IQR: 12–22). Overall prevalence of pN1 was 86/437 (19.7%), including 7/86 (8.1%) suspicious nodes on PET that remained in situ after ePLND. Of the patients with pN1 disease, 22/174 (12.6%) and 63/260 (24.2%) came from the intermediate-risk and high-risk subgroups, respectively. Baseline characteristics for radioligand subgroups are presented in Table S1. As shown, median PSA and distributions of clinical stage, MRI stage and biopsy grade group were comparable. The median number of nodes removed was higher for the [^18^F]F-PSMA-1007 group (21 IQR 16–27 vs. 14 IQR 10–19) (Table 2 and S1).

**Table 2:** Baseline characteristics of the overall population

Characteristics		LNI^−^ (*n* = 351)	LNI^+^ (*n* = 86)
Age (years)	Median (IQR)	66 (61.0–71.0)	67 (62.0–71.0)
PSA (ng/mL)	Median (IQR)	9.6 (6.5–16.2)	11.6 (8.0–19.6)
PSMAvol (cm^3^)	Median (IQR)	4.5 (1.1–10.2)	7.9 (3.5–17.8)
Clinical stage	T1 (%)	171 (48.7)	26 (30.2)
	T2a (%)	105 (29.9)	33 (38.4)
	T2b (%)	27 (7.7)	5 (5.8)
	T2c (%)	5 (1.4)	1 (1.2)
	T3a (%)	37 (10.5)	18 (20.9)
	T3b (%)	6 (1.7)	2 (2.3)
	T4 (%)	0 (0.0)	1 (1.2)
MRI-stage	T1 (%)	12 (3.4)	0 (0.0)
	T2 (%)	188 (53.6)	32 (37.2)
	T3a (%)	116 (33.0)	33 (38.4)
	T3b (%)	34 (9.7)	20 (23.3)
	T4 (%)	1 (0.3)	1 (1.3)
Biopsy Grade Group	1 (%)	14 (4.0)	0 (0)
	2 (%)	88 (25.1)	11 (12.8)
	3 (%)	101 (28.8)	24 (27.9)
	4 (%)	104 (29.6)	22 (25.6)
	5 (%)	44 (12.5)	29 (33.7)
EAU risk group	Low risk	3 (0.9)	0 (0)
	Intermediate risk	152 (43.3)	22 (25.6)
	High risk	196 (55.8)	64 (74.4)
miN	N0 (%)	335 (95.4)	45 (52.3)
	N1 (%)	10 (2.8)	23 (26.7)
	N2 (%)	6 (1.7)	18 (20.9)
No. of cores taken per patient	Median (IQR)	12 (10.0–15.0)	12 (10.0–15.0)
No. of positive cores	Median (IQR)	6 (4.0–8.0)	7 (5.0–10.0)
Biopsy strategy	Systematic only (%)	109 (31.1)	32 (37.2)
	Target only (%)	41 (11.7)	11 (12.8)
	Systematic + target (%)	201 (57.3)	43 (50.0)
No. of removed and examined lymph nodes	Median (IQR)	16 (12.0–22.0)	19 (13.3–25.0)
No. of positive lymph nodes	Median (IQR)	0 (0)	1 (1–2)
Pathologic stage	pT2 (%)	177 (50.4)	10 (11.6)
	pT3a (%)	119 (33.9)	30 (34.9)
	pT3b (%)	53 (15.1)	46 (53.5)
	pT4 (%)	2 (0.6)	0 (0)
Surgical Grade Group	1 (%)	14 (4.0)	0 (0)
	2 (%)	90 (25.6)	11 (12.8)
	3 (%)	102 (29.1)	24 (27.9)
	4 (%)	103 (29.3)	24 (27.9)
	5 (%)	42 (12.0)	27 (31.4)

IQR = interquartile range; MRI = magnetic resonance imaging; PET = positron emission tomography; PSA = prostate specific antigen; PSMAvol = prostate specific membrane antigen volume

*Sum of percentages may not add up to 100% due to rounding

### Diagnostic accuracy of PSMA PET/CT of detecting stage LNI

PSMA PET/CT detected lymph node involvement with a sensitivity of 47.7% (41/86) (95% CI: 36.8–58.7) in patients with pN1 disease across the entire cohort. The PPV was 71.9% (95% CI: 58.5–83.0) and the NPV was 88.2% (95% CI: 84.5–91.2) (Table 3). In the [^68^Ga]Ga-PSMA-11 subset, LNI was detected in 50.0% (24/48) of patients (95% CI: 35.2–64.8), with a PPV of 68.6% (95% CI: 50.7–83.1) and an NPV of 89.3% (95% CI: 84.5–93.0). In the [^18^F]F-PSMA-1007 subset, LNI was detected in 44.7% (17/38) of patients (95% CI: 28.6–61.7), with a PPV of 77.3% (95% CI: 54.6–92.2) and an NPV of 86.5% (95% CI: 80.2–91.5) (Table 3).

**Table 3:** Diagnostic accuracy measures of PSMA PET/CT for the prediction of LNI

	Complete dataset (*n* = 437)		[^68^Ga]Ga-PSMA-11 (*n* = 259)		[^18^F]F-PSMA-1007 (*n* = 178)	
	*n*/*N*	Result, % (95% CI)	*n*/*N*	Result, % (95% CI)	*n*/*N*	Result, % (95% CI)
Sensitivity	41/86	47.7 (36.8–58.7)	24/48	50.0 (35.2–64.8)	17/38	44.7 (28.6–61.7)
Specificity	335/351	95.4 (92.7–97.4)	200/211	94.8 (90.9–97.4)	135/140	96.4 (91.9–98.8)
PPV	41/57	71.9 (58.5–83.0)	24/35	68.6 (50.7–83.1)	17/22	77.3 (54.6–92.2)
NPV	335/380	88.2 (84.5–91.2)	200/224	89.3 (84.5–93.0)	135/156	86.5 (80.2–91.5)

### Model discrimination and calibration in the overall population

Model discrimination, evaluated using the AUC, was as follows: 0.81 (95% CI: 0.76–0.86) for the Amsterdam-Brisbane-Sydney model, 0.79 (95% CI: 0.74–0.85) for Muehlematter Model 1, 0.68 (95% CI: 0.61–0.74) for the MSKCC model, 0.67 (95% CI: 0.61–0.73) for the Briganti 2012 model, 0.67 (95% CI: 0.61–0.73) for the Muehlematter Model 3, and 0.66 (95% CI: 0.59–0.74) for the Muehlematter Model 2 (Table 4).

**Table 4:** Area under the curve of the six models in the overall population and split by radioligand subgroup

Tracer	Model	AUC (95% CI)	AUC (95% CI) in original article
Overall population (*n* = 437)	Amsterdam-Brisbane-Sydney	0.81 (0.76–0.86)	0.81 (0.78–0.85) 0.75 (0.69–0.81)^*^
	Briganti 2012	0.67 (0.61–0.73)	0.88 (not reported)
	Memorial Sloan Kettering Cancer Center	0.68 (0.61–0.74)	0.84 (not reported)
	Muehlematter Model 1	0.79 (0.74–0.85)	0.84 (0.82–0.87) 0.92 (0.86–0.98)^*^
	Muehlematter Model 2	0.66 (0.59–0.74)	0.82 (0.79–0.84)
	Muehlematter Model 3	0.67 (0.61–0.73)	0.72 (0.69–0.75)
[^68^Ga]Ga-PSMA-11 (*n* = 259)	Amsterdam-Brisbane-Sydney	0.82 (0.75–0.89)	-
	Briganti 2012	0.64 (0.56–0.72)	-
	Memorial Sloan Kettering Cancer Center	0.67 (0.59–0.75)	-
	Muehlematter Model 1	0.78 (0.70–0.85)	-
	Muehlematter Model 2	0.68 (0.57–0.79)	-
	Muehlematter Model 3	0.66 (0.58–0.74)	-
[^18^F]F-PSMA-1007 (*n* = 178)	Amsterdam-Brisbane-Sydney	0.80 (0.72–0.88)	-
	Briganti 2012	0.72 (0.62–0.81)	-
	Memorial Sloan Kettering Cancer Center	0.69 (0.58–0.79)	-
	Muehlematter Model 1	0.81 (0.73–0.89)	-
	Muehlematter Model 2	0.64 (0.52–0.75)	-
	Muehlematter Model 3	0.70 (0.60–0.79)	-

*Values originate from the results of the external validation in the original paper

**Fig. 1:**
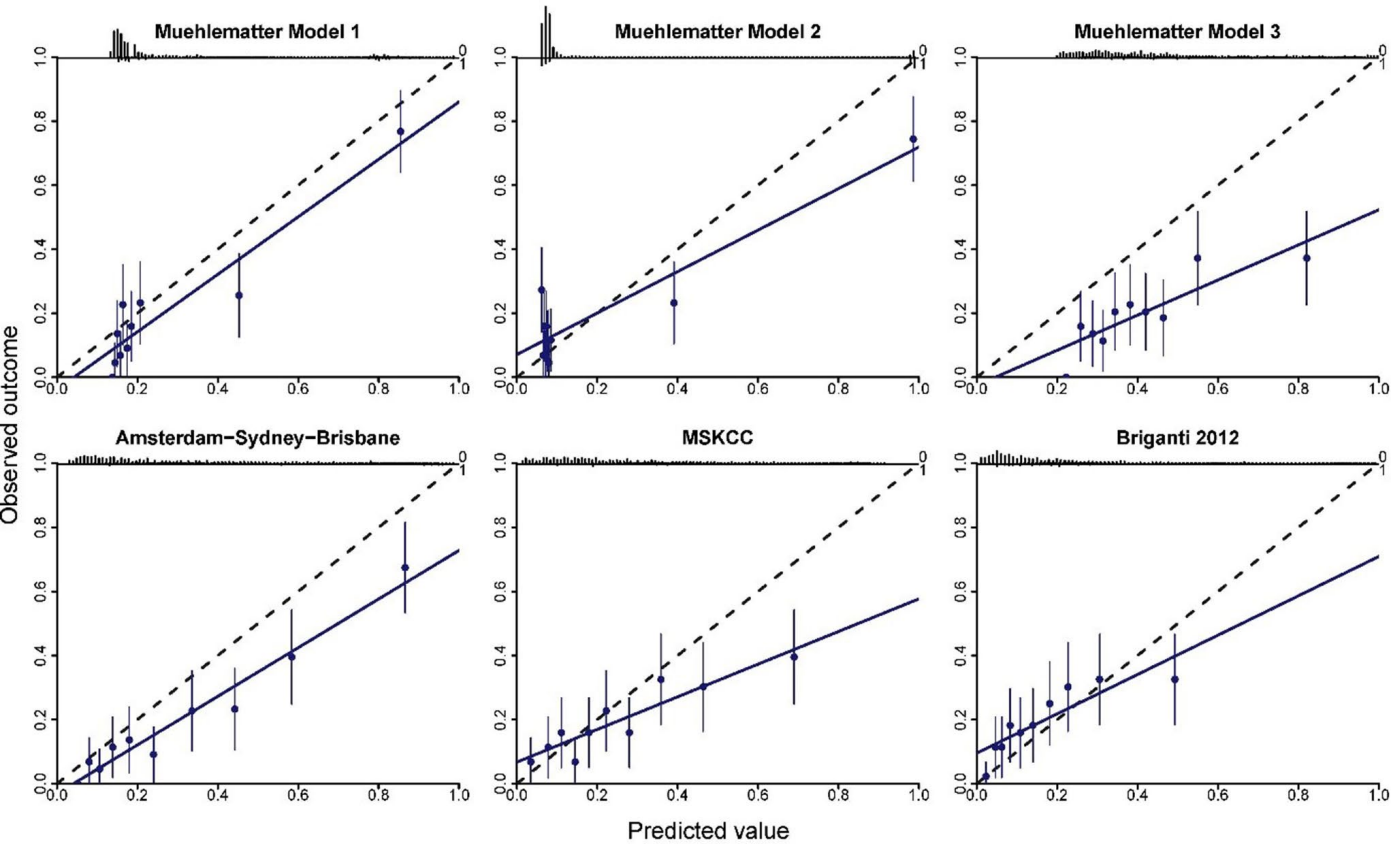
Model calibration plots of predicted probability versus observed probability of lymph node involvement for the Muehlematter models 1 to 3, the Amsterdam-Sydney-Brisbane model, the MSKCC Pre-Radical Prostatectomy nomogram with cores, and the Briganti 2012 nomogram. The dashed lines represent a straight fit, the blue line denotes the calibration curve, the ten blue dots denote tenths of the patient cohort (95% CI). The black bars at the top represent the distribution of predicted probabilities

For calibration, Muehlematter Model 1 demonstrated good agreement between predicted and observed risks, with slight overestimation (Fig. 1). The Amsterdam-Brisbane-Sydney nomogram showed moderate agreement, with more pronounced overestimation across all thresholds. The MSKCC and Briganti 2012 models exhibited moderate agreement, though overestimation was observed at threshold probabilities > 40%. In contrast, Muehlematter Model 2 and Muehlematter Model 3 showed poor calibration across all risk thresholds (Fig. 1).

### Systematic evaluation of rates of LNI missed and detected for clinically relevant thresholds

A systematic analysis of the event status of patients who fall above and below the risk thresholds between 0% and 30% for Muehlematter Model 1 is provided in Table S2. Using risk thresholds of 15% and 16% could avoid ePLND in 71 (16.2%) and 144 (33.0%) patients, while missing respectively 0% and 9/86 (10.5%) of patients with LNI (Table S2). For the Amsterdam-Brisbane-Sydney model, using a threshold of 8% and 11% could spare ePLNDs in respectively 65 (14.9%) and 115 (26.3%) of patients, while missing 0% and 4/86 (4.7%) of patients with LNI (Table S3). For the Briganti 2012 and MSKCC, using thresholds of 5% and 7% would spare ePLND in respectively 75 (17.2%) and 128 (29.3%) and 37 (8.5%) and 56 (12.8%), while missing LNI in 4 (4.7%) and 11 (12.8%) and 2 (2.3%) and 4 (4.7%), respectively (Tables S4 and S5).

### Net benefit determined by decision curve analysis

**Fig. 2:**
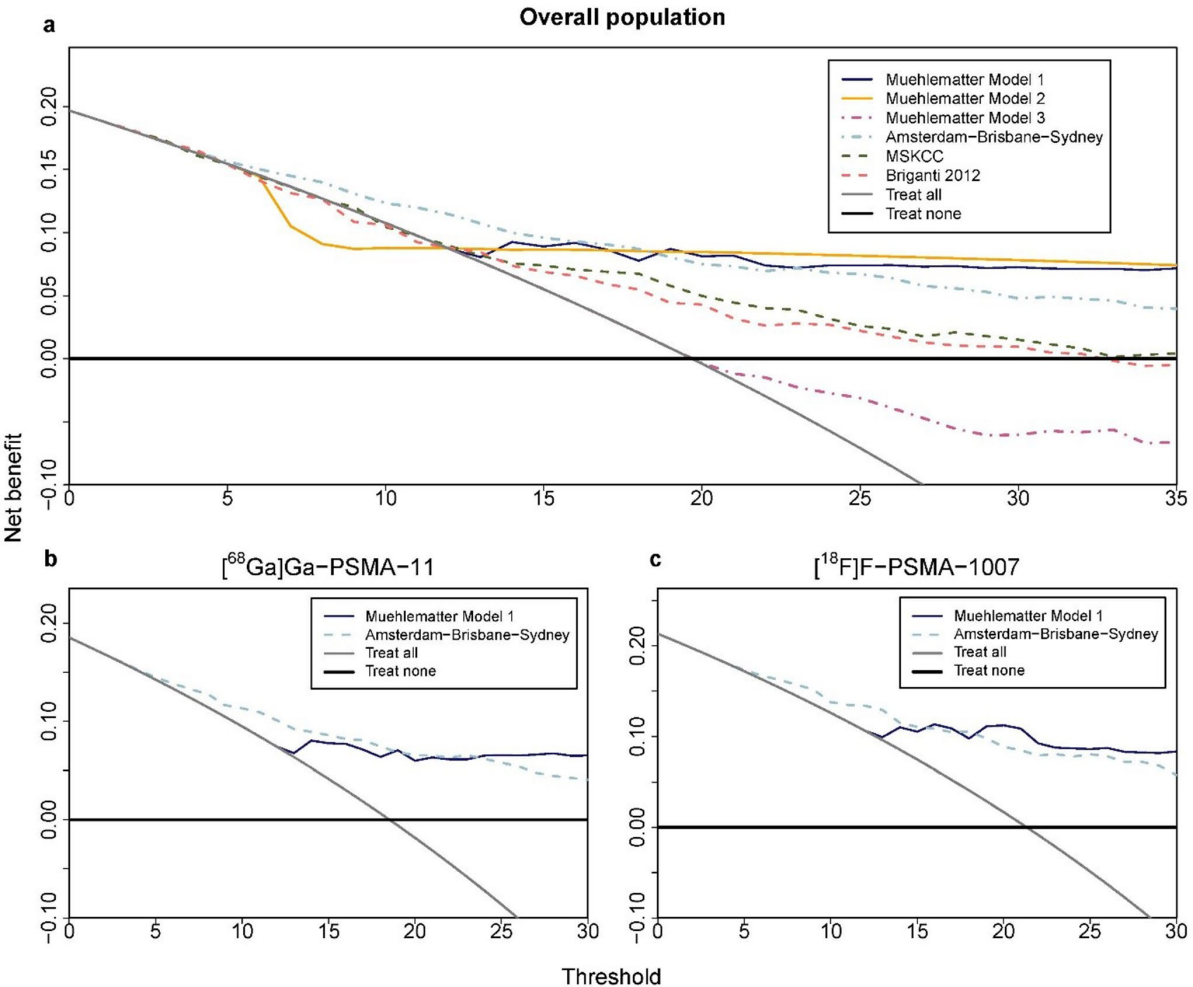
Decision curves of all six models (Muehlematter Models 1 to 3; Amsterdam-Brisbane-Sydney; MSKCC; Briganti 2012) depicting the net benefit (NB, y-axis) of a model or a strategy (treat-all or treat-none with ePLND) according to a risk threshold (x-axis) in the overall population (**a**), and by radioligand [^68^Ga]Ga-PSMA-11 (**b**), or [^18^F]F-PSMA-1007 (**c**) for the two nomograms with the highest net benefit

Decision curve analysis (DCA) demonstrated that the Amsterdam-Brisbane-Sydney model provided superior net benefit compared to the “treat all” approach and other models across risk thresholds of 5–20%. Muehlematter Model 1 exhibited favorable net benefit specifically within the 14–20% threshold range when compared to the “treat all” strategy (Fig. 2a). Both the MSKCC and Briganti 2012 models demonstrated comparable net benefit, exceeding that of the ‘treat all’ approach across thresholds from 11 to 25%.

### Subgroup analysis of model performance for Muehlematter model 1 and the Amsterdam-Brisbane-Sydney nomograms in evaluating [^18^F]F-PSMA-1007 and [^68^Ga]Ga-PSMA-11

A total of 178 patients (40.7%) underwent [^18^F]F-PSMA-1007 PET, while 259 patients (59.3%) underwent [^68^Ga]Ga-PSMA-11 PET (Table S1). Decision curve analysis in the tracer subgroups indicated that both models exceeded the net benefit of the “treat all” and “treat none” strategies. The Amsterdam-Brisbane-Sydney model showed clinical benefit for risk thresholds of 5–35%, while Muehlematter Model 1 was optimal for thresholds between 14% and 30% (Fig. 2b, c).

**Fig. 3:**
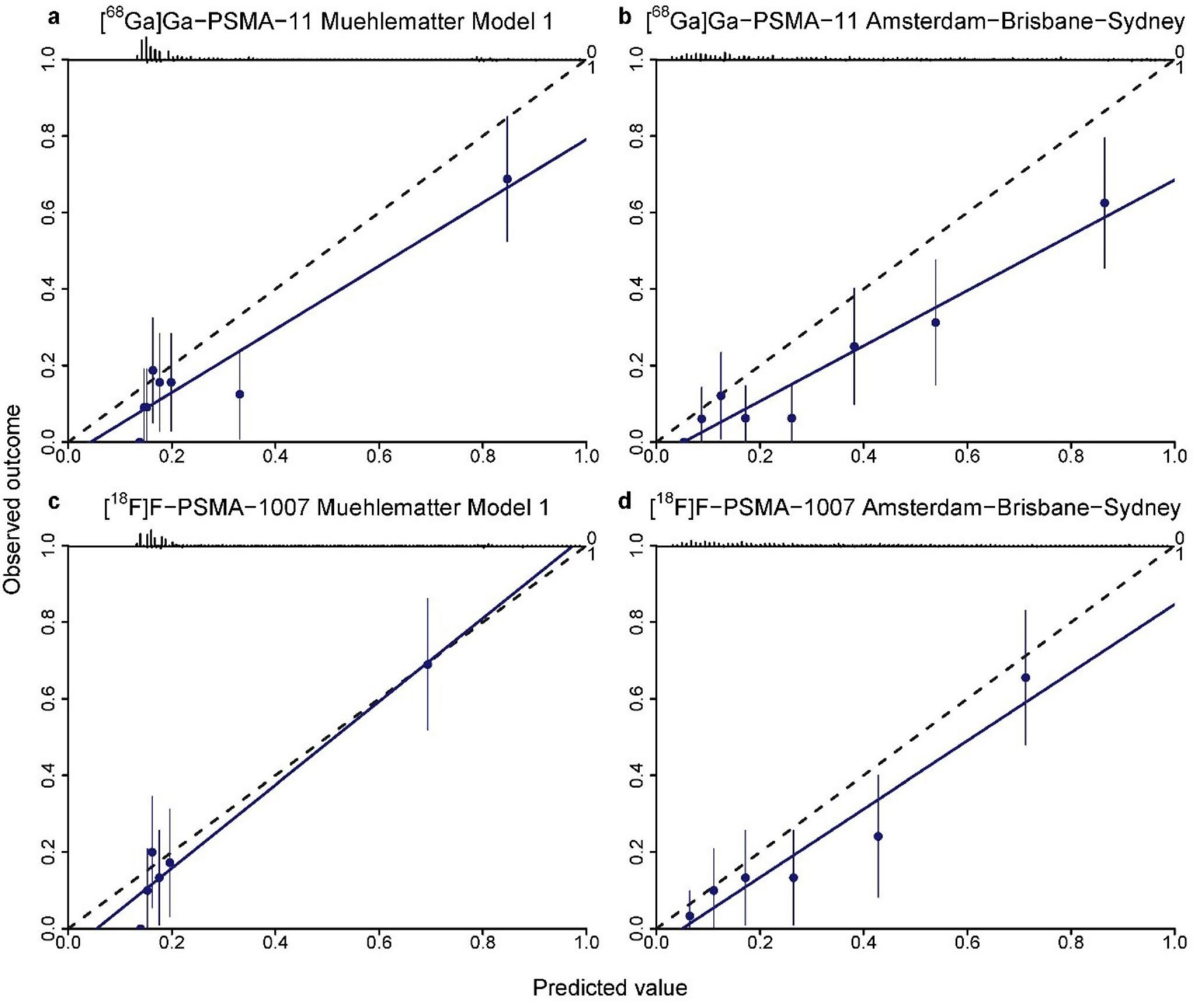
Model calibration plots of predicted probability versus observed probability of lymph node involvement for the Muehlematter Model 1 (**a**, **c**) and the Amsterdam-Sydney-Brisbane model (**b**, **d**) in the [^68^Ga]Ga-PSMA-11 (**a**, **b**) and the [^18^F]F-PSMA-1007 (**c**, **d**) subsets. The dashed lines represent a straight fit, the blue line denotes the calibration curve, the eight blue dots denote eights of the patient cohort (95% CI). The black bars at the top represent the distribution of predicted probabilities

Both the Muehlematter Model 1 and Amsterdam-Brisbane-Sydney nomograms showed superior calibration in the [^18^F]F-PSMA-1007 group compared to the [^68^Ga]Ga-PSMA-11 subgroup (Fig. 3). In both cohorts, systematic overestimation of the predicted risk was observed, except for Muehlematter Model 1 in the [^18^F]F-PSMA-1007 subset, showing slight overestimation for predicted probabilities (> 80%). For discrimination, Muehlematter Model 1 showed a higher AUC in the [^18^F]F-PSMA-1007 cohort (AUC 0.81, 95% CI: 0.73–0.89) compared to the [^68^Ga]Ga-PSMA-11 cohort (AUC 0.78, 95% CI: 0.70–0.85) (Table 4). Conversely, the Amsterdam-Brisbane-Sydney model performed slightly better in the [^68^Ga]Ga-PSMA-11 cohort (AUC 0.82, 95% CI: 0.75–0.89) than in the [^18^F]F-PSMA-1007 cohort (AUC 0.80, 95% CI: 0.72–0.88).

### Subgroup analysis of model performance including patients staged miN0

The AUCs observed in the miN0 cohort (*N* = 380) were 0.70 (95% CI: 0.63–0.78) for the Amsterdam-Brisbane-Sydney model and 0.64 (95% CI: 0.57–0.72) for Muehlematter Model 1 (Table S6). The MSKCC model demonstrated the highest AUC of 0.72 (95% CI: 0.65–0.80). The AUCs for Muehlematter Models 2 and 3 and the Briganti 2012 model were 0.61 (95% CI: 0.52–0.70), 0.64 (95% CI: 0.56–0.71), and 0.68 (95% CI: 0.60–0.76), respectively. Compared to the overall cohort, all models—except for Muehlematter Model 1—exhibited a decrease in agreement between predicted and observed probabilities (Fig. S1). The highest net benefit in this subgroup was observed for the Briganti 2012 model, which outperformed all other models for risk thresholds between 10% and 20% (Fig. S2).

## Discussion

In this study an external validation of nomograms incorporating PSMA PET data for LNI was performed in a multicenter, international cohort of patients who underwent RARP and ePLND following preoperative staging with PSMA PET. Both the Amsterdam-Brisbane-Sydney model and Muehlematter Model 1 demonstrated strong discriminatory performance, achieving an AUC of 0.81 and 0.79, respectively, outperforming the MSKCC, Briganti 2012, and the two remaining Muehlematter (Model 2 and 3) models, which had AUCs of 0.67, and 0.68, 0.66, and 0.67, respectively. Furthermore, both calibration and net benefit analyses favored the Amsterdam-Brisbane-Sydney and Muehlematter Model 1.

Employing a conservative approach to avoid missing any LNI cases, applying thresholds of 8% for the Amsterdam-Brisbane-Sydney nomogram and 15% for Muehlematter Model 1 could spare ePLND in 15% and 16% of patients, respectively, without missing any LNI-positive cases. Using a higher threshold of 11%, the Amsterdam-Brisbane-Sydney model could spare 26.3% of the PLNDs at the cost of missing 4.7% of LNI cases. This underscores the potential for refining ePLND indications of these tools in patients preoperatively staged with PSMA PET, based on individual patient characteristics and preferences. In comparison, to achieve similar rates of spared ePLND using the MSKCC and Briganti 2012 nomograms, an 8% threshold with MSKCC would spare 16% of patients while missing 7% of LNI cases, and a 5% threshold with Briganti 2012 would spare 17% of patients while missing 4.7% of LNI cases. It should be noted that the range of predicted probabilities differs between nomograms. The distribution of Muehlematter Model 1 is narrower (ranging from 13.0 to 99.0%) compared to that of the Amsterdam-Brisbane-Sydney model (2.7–99.0%), which is consistent with the distribution observed in the original study [4]. These differences should be considered when establishing a threshold value for clinical decision-making. Nevertheless, our external validation confirms the favorable utility of models incorporating PSMA PET information, as previously reported in their respective development studies [21, 22].

In this study, we evaluated a broad population of PSMA PET staged patients undergoing RARP and ePLND, including patients with miN1-2 disease. Inclusion of patients with miN1-2 disease may explain the more favorable performance observed for the Amsterdam-Brisbane-Sydney tool, as this nomogram was developed on a cohort also including these patients. Additionally, based on the distribution of coefficients, miN1 status is a key determinant of predicted LNI risk in both the Amsterdam-Brisbane-Sydney and Muehlematter 1 nomograms [21, 22]. Other important contemporary predictors for LNI, all included in the Amsterdam-Brisbane-Sydney and Briganti 2019 and Briganti 2023 nomograms, are MRI T stage, percentage of positive systematic biopsy cores and biopsy GG. Multivariable logistic regression analysis using backward elimination with current study data identified these four predictors as the strongest factors associated with LNI risk prediction. These factors outperformed PSMA_vol_, serum PSA, age, and clinical stage assessed by DRE (Supplementary Table S7), suggesting their superior prognostic relevance.

Our findings are complimentary to those of other external validation studies, revealing that available clinical nomograms predicting LNI had suboptimal performance in patients staged miN0M0 [30, 31]. The very recent external validation study showed that the Briganti 2023 outperformed other available tools, including the Briganti 2019 and the Amsterdam-Brisbane-Sydney nomograms, for this patient subgroup [31]. The Briganti 2023 nomogram is specifically developed for patients with miN0M0 disease, and includes biopsy GG, clinical stage at mpMRI, maximum diameter of the index lesion, preoperative PSA and percentage of positive cores at systematic biopsy [30]. The fact that this nomogram outperformed other tools that were not specifically designed for miN0M0 populations highlights the importance of using models tailored to the target population. Apart from the fact that a portion of the patients in the current cohort was also included in the development of both the Briganti 2019 and Briganti 2023 nomograms, not all patients underwent the biopsy strategies required by these nomograms. Therefore, this cohort was unfortunately not suitable for a reliable external validation of these tools.

However, our subanalysis, which included only patients staged as miN0, revealed that the performance of both the Amsterdam-Brisbane-Sydney and Muehlematter 1 nomograms was substantially lower in this subset. In clinical practice, patients opting for surgery with miN1-2 would in the vast majority of cases also undergo ePLND. This is supported by the overall data of this multicenter project including patients preoperatively staged with PSMA PET, treated with RARP with or without ePLND. Among all patients staged as miN1-2, only in one patient (1.4%) ePLND was omitted, which was due to perioperative complications. To conclude, since nomogram-based predicted risk would mostly drive treatment decisions in patients staged miN0, the clinical relevance of both Muehlematter 1 and Amsterdam-Brisbane-Sydney could be suboptimal in their current forms. We would therefore advise to update both of these tools specifically for miN0 patients, to improve their clinical utility in this patient subgroup.

In addition, the generally high reported specificity of PSMA PET for the detection of pelvic nodal metastasis, mostly exceeding 90% in prior studies, it can be further argued if nomogram-based risk calculation provides added value in cases of miN1-2 disease [2, 3, 5]. Based on the observed PPV of 71.9% in this cohort, which aligns with prior studies reporting PPVs between 70% and 81%, incorporating additional clinical prognostic factors may however enhance risk stratification and reduce potential overtreatment. This may be particularly relevant for patients meeting intermediate-risk criteria with miN1-2 findings on PSMA PET, as the observed PPV in the intermediate-risk group for detecting LNI was 58.8%, compared to the high-risk group, where a PPV of 77.5% was observed (Tables S8 and S9).

A notable strength of this study is the validation of the [^68^Ga]Ga-PSMA-11 PET–based Muehlematter Model 1 nomogram using [^18^F]F-PSMA-1007 PET data. Given that this nomogram incorporates the SUV-based quantitative parameter PSMA_vol_, our findings suggest that the nomogram’s predictive accuracy is maintained regardless of the specific PSMA inhibitor used (PSMA-11 vs- PSMA-1007) and regardless the specific isotope (68Ga vs. 18F). This indicates potential interchangeability between different PSMA-PET techniques in this predictive model. Other factors that may have contributed to the favorable findings in the [^18^F]F-PSMA-1007 cohort included higher median number of resected nodes (21 vs. 14) and lower biopsy downgrading rates (31% vs. 38%) compared with the [^68^Ga]Ga-PSMA-11 subgroup (Tables S1, S10–S12). Additionally, the majority (93.6%) of patients staged with [^18^F]F-PSMA-1007 were treated at a single institution, which also may partially explain the favorable fit observed.

Since the introduction of PSMA PET for primary staging, the detection rates of both pelvic nodal lesions and distant metastases have significantly influenced treatment selection strategies [32, 33]. Despite its advantages, with a sensitivity of only 40–50% for detecting LNI, ePLND remains the most accurate method currently available for nodal staging. While incorporating PSMA PET findings into clinical prediction models may improve patient selection, our systematic analysis of LNIs detected and missed suggests ePLND will still need to be performed in a substantial number of node-negative patients to ensure that clinically significant rates of node-positive cases are not missed.

A critical question remains whether ePLND can be omitted in all patients undergoing radical prostatectomy with preoperative miN0M0 PET findings. This uncertainty is particularly significant as the undetected nodal lesions are typically small, millimetric in size, and their impact on long-term outcomes remains unclear [34]. Notably, PSMA PET-guided metastasis-directed therapy in the recurrence setting could serve as a reliable safety net, potentially mitigating the consequences of undetected nodal disease. The results of the ongoing Dutch national randomised controlled trial, PSMA SELECT, are highly anticipated. This trial randomised patients with an LNI risk > 5% to either a nomogram-based ePLND approach (ePLND performed universally) or a PSMA PET-guided strategy (ePLND omitted in miN0 patients and performed only in miN1 patients) [35]. These findings are expected to provide important insights into the optimal integration of PSMA PET in primary staging and treatment planning.

This study has a number of limitations. First, only the highest biopsy ISUP GG per prostate lobe were available in this study; and all positive cores on the ipsilateral side were regarded as ISUP GG ≥ 2 in case this was also the highest ipsilateral GG reported. This could have led to overestimation of the predicted probabilities for the Amsterdam-Brisbane-Sydney nomogram. However, a sensitivity analysis performed, counting a maximum number of 1 positive core per prostate lobe with GG ≥ 2 did not alter the study’s conclusions (Tables S13 and S14, Figs. S3 and S4). Second, although all patients underwent ePLND, encompassing the obturator fossa and the internal and external iliac arteries, variations in surgical templates were permitted across different hospitals and surgeons. Unfortunately, rates of template modifications were not available, which could limit the generalizability of our findings. Third, there was no central review of MRI, PSMA PET, or histopathological evaluation. However, inter-reader variability of these parameters can also be seen as a strength, as it enables more robust estimates and reflects real-world clinical practice. Fourth, among the 86 patients classified as pN1, 7 (8.1%) were classified as pN1 based on PSA persistence and presence of the suspicious node on postoperative PSMA PET. The lack of histopathological confirmation in these patients should be considered as a limitation. Fifth, the unavailability of certain parameters included in the Briganti 2017, Briganti 2019 and Briganti 2023 nomograms prevented their external validation in this study [16, 30, 36].

Lastly, the present cohort consisted of selected cases for both PSMA PET and ePLND, which could have introduced selection bias.

## Conclusion

External validation of Muehlematter Model 1 and Amsterdam-Brisbane-Sydney nomograms for predicting LNI demonstrated their strong model discrimination, moderate calibration, and good clinical utility. Both nomograms outperformed the PSMA PET-naïve nomograms, Briganti 2012 and MSKCC, within this patient population. Notably, their performance remained consistent regardless of the radioligand used ([^68^Ga]Ga-PSMA-11 PET/CT or [^18^F]F-PSMA-1007). These findings support their reliability as tools to guide clinical decision-making effectively.

## Electronic supplementary material

Below is the link to the electronic supplementary material.

Supplementary Material 1

## Data Availability

The datasets generated during and/or analyzed during the current study are available from the corresponding author on reasonable request.
