# Supplementary material for: External validation of nomograms including PSMA PET information for the prediction of lymph node involvement of prostate cancer
Source: Eur J Nucl Med Mol Imaging. 2025 Apr 2;52(10):3744–56. doi: 10.1007/s00259-025-07241-y (PMC12316820; doi:10.1007/s00259-025-07241-y)
Supplement: Supplementary file 1 — Supplementary Material 1 [file 259_2025_7241_MOESM1_ESM.docx]

**Supplementary**

**Table S1** Baseline characteristics of the [^68^Ga]Ga-PSMA-1 and [^18^F]F-PSMA-1007 PET/CT subgroups

| Characteristics | | Gallium (n = 259) | Fluor (n = 178) |
| --- | --- | --- | --- |
| Age (years) | Median (IQR) | 66 (61.0 - 71.0) | 68 (63.0 - 71.0) |
| PSA (ng/mL) | Median (IQR) | 9.9 (6.6 - 18.8) | 10.1 (6.9 - 15.7) |
| PSMAvol (cm3) | Median (IQR) | 4.3 (1.1 - 10.7) | 6.2 (2.9 - 11.2) |
| Clinical stage | T1 (%) | 117 (45.2) | 80 (44.9) |
|  | T2a (%) | 73 (28.2) | 65 (36.5) |
|  | T2b (%) | 23 (8.9) | 9 (5.1) |
|  | T2c (%) | 5 (1.9) | 1 (0.6) |
|  | T3a (%) | 32 (12.4) | 23 (12.9) |
|  | T3b (%) | 8 (3.1) | 0 (0.0) |
|  | T4 (%) | 1 (0.4) | 0 (0.0) |
| MRI-stage | T1 | 5 (1.9) | 7 (3.9) |
|  | T2 | 134 (51.7) | 86 (48.3) |
|  | T3a | 86 (33.2) | 63 (35.4) |
|  | T3b | 33 (12.7) | 21 (11.8) |
|  | T4 | 1 (0.4) | 1 (0.6) |
| Grade Group | 1 | 5 (1.9) | 9 (5.1) |
|  | 2 | 59 (22.8) | 40 (22.5) |
|  | 3 | 70 (27.0) | 55 (30.9) |
|  | 4 | 78 (30.1) | 48 (27.0) |
|  | 5 | 47 (18.1) | 26 (14.6) |
| No. of cores taken per patient | Median (IQR) | 12 (10.0 - 16.0) | 12 (10.0 - 15.0) |
| No. of positive cores | Median (IQR) | 6.0 (4.0 – 9.0) | 6.0 (4.0 - 9.0) |
| No. of removed and examined lymph nodes | Median (IQR) | 14 (10.0 - 19.0) | 21 (16.0 - 27.0) |
| EAU risk group | Low risk | 2 (0.8) | 1 (0.6) |
|  | Intermediate risk | 95 (36.7) | 79 (44.4) |
|  | High risk | 162 (62.5) | 98 (55.1) |
| PET N status | N0 | 224 (86.5) | 156 (87.6) |
|  | N1 | 35 (13.5) | 22 (12.4) |
| pN | pN0 | 211 (81.5) | 140 (78.7) |
|  | pN1 | 48 (18.5) | 38 (21.3) |
| IQR = interquartile range; MRI = magnetic resonance imaging; PET = positron emission tomography; PSA = prostate-specific antigen; PSMAvol = prostate-specific membrane antigen volume  *Sum of percentages may not add up to 100% due to rounding | | | |

**Table S2** Analyses of the **Muehlematter Model 1** model cut-offs used to discriminate between patients with or without lymph node involvement confirmed at extended pelvic lymph node dissection

| Calculated probabil-  ity of LNI % (cut-off) | Number of patients, n (%) | | | | | | | Sensitivity | Specificity |
| --- | --- | --- | --- | --- | --- | --- | --- | --- | --- |
|  | Below the cut-off (ePLND not recommended) | | |  | Equal to or above the cut-off (ePLND recommended) | | |  |  |
|  | Total | Without LNI | With LNI |  | Total | Without LNI | With LNI |  |  |
| 0 | 0 (0) | 0 (0) | 0 (0) |  | 437 (100) | 351 (100) | 86 (100) | 1.000 | 0.000 |
| 1 | 0 (0) | 0 (0) | 0 (0) |  | 437 (100) | 351 (100) | 86 (100) | 1.000 | 0.000 |
| 2 | 0 (0) | 0 (0) | 0 (0) |  | 437 (100) | 351 (100) | 86 (100) | 1.000 | 0.000 |
| 3 | 0 (0) | 0 (0) | 0 (0) |  | 437 (100) | 351 (100) | 86 (100) | 1.000 | 0.000 |
| 4 | 0 (0) | 0 (0) | 0 (0) |  | 437 (100) | 351 (100) | 86 (100) | 1.000 | 0.000 |
| 5 | 0 (0) | 0 (0) | 0 (0) |  | 437 (100) | 351 (100) | 86 (100) | 1.000 | 0.000 |
| 6 | 0 (0) | 0 (0) | 0 (0) |  | 437 (100) | 351 (100) | 86 (100) | 1.000 | 0.000 |
| 7 | 0 (0) | 0 (0) | 0 (0) |  | 437 (100) | 351 (100) | 86 (100) | 1.000 | 0.000 |
| 8 | 0 (0) | 0 (0) | 0 (0) |  | 437 (100) | 351 (100) | 86 (100) | 1.000 | 0.000 |
| 9 | 0 (0) | 0 (0) | 0 (0) |  | 437 (100) | 351 (100) | 86 (100) | 1.000 | 0.000 |
| 10 | 0 (0) | 0 (0) | 0 (0) |  | 437 (100) | 351 (100) | 86 (100) | 1.000 | 0.000 |
| 11 | 0 (0) | 0 (0) | 0 (0) |  | 437 (100) | 351 (100) | 86 (100) | 1.000 | 0.000 |
| 12 | 0 (0) | 0 (0) | 0 (0) |  | 437 (100) | 351 (100) | 86 (100) | 1.000 | 0.000 |
| 13 | 0 (0) | 0 (0) | 0 (0) |  | 437 (100) | 351 (100) | 86 (100) | 1.000 | 0.000 |
| 14 | 11 (2.5) | 11 (3.1) | 0 (0) |  | 426 (97.5) | 340 (96.9) | 86 (100) | 1.000 | 0.031 |
| **15** | 71 (16.2) | 71 (20.2) | 0 (0) |  | 366 (83.8) | 280 (79.8) | 86 (100) | 1.000 | 0.202 |
| **16** | 144 (33) | 135 (38.5) | 9 (10.5) |  | 293 (67) | 216 (61.5) | 77 (89.5) | 0.895 | 0.385 |
| 17 | 204 (46.7) | 189 (53.8) | 15 (17.4) |  | 233 (53.3) | 162 (46.2) | 71 (82.6) | 0.826 | 0.538 |
| 18 | 245 (56.1) | 223 (63.5) | 22 (25.6) |  | 192 (43.9) | 128 (36.5) | 64 (74.4) | 0.744 | 0.635 |
| 19 | 286 (65.4) | 255 (72.6) | 31 (36) |  | 151 (34.6) | 96 (27.4) | 55 (64) | 0.640 | 0.726 |
| 20 | 315 (72.1) | 283 (80.6) | 32 (37.2) |  | 122 (27.9) | 68 (19.4) | 54 (62.8) | 0.628 | 0.806 |
| 21 | 329 (75.3) | 293 (83.5) | 36 (41.9) |  | 108 (24.7) | 58 (16.5) | 50 (58.1) | 0.581 | 0.835 |
| 22 | 338 (77.3) | 301 (85.8) | 37 (43) |  | 99 (22.7) | 50 (14.2) | 49 (57) | 0.570 | 0.858 |
| 23 | 347 (79.4) | 306 (87.2) | 41 (47.7) |  | 90 (20.6) | 45 (12.8) | 45 (52.3) | 0.523 | 0.872 |
| 24 | 351 (80.3) | 309 (88) | 42 (48.8) |  | 86 (19.7) | 42 (12) | 44 (51.2) | 0.512 | 0.880 |
| 25 | 356 (81.5) | 314 (89.5) | 42 (48.8) |  | 81 (18.5) | 37 (10.5) | 44 (51.2) | 0.512 | 0.895 |
| 26 | 358 (81.9) | 316 (90) | 42 (48.8) |  | 79 (18.1) | 35 (10) | 44 (51.2) | 0.512 | 0.900 |
| 27 | 360 (82.4) | 318 (90.6) | 42 (48.8) |  | 77 (17.6) | 33 (9.4) | 44 (51.2) | 0.512 | 0.906 |
| 28 | 364 (83.3) | 321 (91.5) | 43 (50) |  | 73 (16.7) | 30 (8.5) | 43 (50) | 0.500 | 0.915 |
| 29 | 366 (83.8) | 323 (92) | 43 (50) |  | 71 (16.2) | 28 (8) | 43 (50) | 0.500 | 0.920 |
| 30 | 369 (84.4) | 325 (92.6) | 44 (51.2) |  | 68 (15.6) | 26 (7.4) | 42 (48.8) | 0.488 | 0.926 |

**Table S3** Analyses of the **Amsterdam-Brisbane-Sydney** model cut-offs used to discriminate between patients with or without lymph node involvement confirmed at extended pelvic lymph node dissection

| Calculated probabil-  ity of LNI % (cut-off) | Number of patients, n (%) | | | | | | | Sensitivity | Specificity |
| --- | --- | --- | --- | --- | --- | --- | --- | --- | --- |
|  | Below the cut-off (ePLND not recommended) | | |  | Equal to or above the cut-off (ePLND recommended) | | |  |  |
|  | Total | Without LNI | With LNI |  | Total | Without LNI | With LNI |  |  |
| 0 | 0 (0) | 0 (0) | 0 (0) |  | 437 (100) | 351 (100) | 86 (100) | 1.000 | 0.000 |
| 1 | 0 (0) | 0 (0) | 0 (0) |  | 437 (100) | 351 (100) | 86 (100) | 1.000 | 0.000 |
| 2 | 0 (0) | 0 (0) | 0 (0) |  | 437 (100) | 351 (100) | 86 (100) | 1.000 | 0.000 |
| 3 | 6 (1.4) | 6 (1.7) | 0 (0) |  | 431 (98.6) | 345 (98.3) | 86 (100) | 1.000 | 0.017 |
| 4 | 7 (1.6) | 7 (2) | 0 (0) |  | 430 (98.4) | 344 (98) | 86 (100) | 1.000 | 0.02 |
| 5 | 16 (3.7) | 16 (4.6) | 0 (0) |  | 421 (96.3) | 335 (95.4) | 86 (100) | 1.000 | 0.046 |
| 6 | 32 (7.3) | 32 (9.1) | 0 (0) |  | 405 (92.7) | 319 (90.9) | 86 (100) | 1.000 | 0.091 |
| 7 | 50 (11.4) | 50 (14.2) | 0 (0) |  | 387 (88.6) | 301 (85.8) | 86 (100) | 1.000 | 0.142 |
| **8** | 65 (14.9) | 65 (18.5) | 0 (0) |  | 372 (85.1) | 286 (81.5) | 86 (100) | 1.000 | 0.185 |
| 9 | 82 (18.8) | 80 (22.8) | 2 (2.3) |  | 355 (81.2) | 271 (77.2) | 84 (97.7) | 0.977 | 0.228 |
| 10 | 102 (23.3) | 98 (27.9) | 4 (4.7) |  | 335 (76.7) | 253 (72.1) | 82 (95.3) | 0.953 | 0.279 |
| **11** | 115 (26.3) | 111 (31.6) | 4 (4.7) |  | 322 (73.7) | 240 (68.4) | 82 (95.3) | 0.953 | 0.316 |
| 12 | 129 (29.5) | 124 (35.3) | 5 (5.8) |  | 308 (70.5) | 227 (64.7) | 81 (94.2) | 0.942 | 0.353 |
| 13 | 143 (32.7) | 136 (38.7) | 7 (8.1) |  | 294 (67.3) | 215 (61.3) | 79 (91.9) | 0.919 | 0.387 |
| 14 | 162 (37.1) | 152 (43.3) | 10 (11.6) |  | 275 (62.9) | 199 (56.7) | 76 (88.4) | 0.884 | 0.433 |
| 15 | 168 (38.4) | 158 (45) | 10 (11.6) |  | 269 (61.6) | 193 (55) | 76 (88.4) | 0.884 | 0.450 |
| 16 | 175 (40) | 165 (47) | 10 (11.6) |  | 262 (60) | 186 (53) | 76 (88.4) | 0.884 | 0.470 |
| 17 | 189 (43.2) | 178 (50.7) | 11 (12.8) |  | 248 (56.8) | 173 (49.3) | 75 (87.2) | 0.872 | 0.507 |
| 18 | 199 (45.5) | 187 (53.3) | 12 (14) |  | 238 (54.5) | 164 (46.7) | 74 (86) | 0.860 | 0.533 |
| 19 | 208 (47.6) | 194 (55.3) | 14 (16.3) |  | 229 (52.4) | 157 (44.7) | 72 (83.7) | 0.837 | 0.553 |
| 20 | 213 (48.7) | 198 (56.4) | 15 (17.4) |  | 224 (51.3) | 153 (43.6) | 71 (82.6) | 0.826 | 0.564 |
| 21 | 224 (51.3) | 208 (59.3) | 16 (18.6) |  | 213 (48.7) | 143 (40.7) | 70 (81.4) | 0.814 | 0.593 |
| 22 | 231 (52.9) | 214 (61) | 17 (19.8) |  | 206 (47.1) | 137 (39) | 69 (80.2) | 0.802 | 0.610 |
| 23 | 242 (55.4) | 225 (64.1) | 17 (19.8) |  | 195 (44.6) | 126 (35.9) | 69 (80.2) | 0.802 | 0.641 |
| 24 | 244 (55.8) | 227 (64.7) | 17 (19.8) |  | 193 (44.2) | 124 (35.3) | 69 (80.2) | 0.802 | 0.647 |
| 25 | 249 (57) | 232 (66.1) | 17 (19.8) |  | 188 (43) | 119 (33.9) | 69 (80.2) | 0.802 | 0.661 |
| 26 | 251 (57.4) | 234 (66.7) | 17 (19.8) |  | 186 (42.6) | 117 (33.3) | 69 (80.2) | 0.802 | 0.667 |
| 27 | 257 (58.8) | 238 (67.8) | 19 (22.1) |  | 180 (41.2) | 113 (32.2) | 67 (77.9) | 0.779 | 0.678 |
| 28 | 264 (60.4) | 244 (69.5) | 20 (23.3) |  | 173 (39.6) | 107 (30.5) | 66 (76.7) | 0.767 | 0.695 |
| 29 | 266 (60.9) | 246 (70.1) | 20 (23.3) |  | 171 (39.1) | 105 (29.9) | 66 (76.7) | 0.767 | 0.701 |
| 30 | 269 (61.6) | 248 (70.7) | 21 (24.4) |  | 168 (38.4) | 103 (29.3) | 65 (75.6) | 0.756 | 0.707 |

**Table S4** Analyses of the **Briganti 2012** model cut-offs used to discriminate between patients with or without lymph node involvement confirmed at extended pelvic lymph node dissection

| Calculated probabil-  ity of LNI % (cut-off) | Number of patients, n (%) | | | | | | | Sensitivity | Specificity |
| --- | --- | --- | --- | --- | --- | --- | --- | --- | --- |
|  | Below the cut-off (ePLND not recommended) | | |  | Equal to or above the cut-off (ePLND recommended) | | |  |  |
|  | Total | Without LNI | With LNI |  | Total | Without LNI | With LNI |  |  |
| 0 | 0 (0) | 0 (0) | 0 (0) |  | 437 (100) | 351 (100) | 86 (100) | 1.000 | 0.000 |
| 1 | 2 (0.5) | 2 (0.6) | 0 (0) |  | 435 (99.5) | 349 (99.4) | 86 (100) | 1.000 | 0.006 |
| 2 | 18 (4.1) | 18 (5.1) | 0 (0) |  | 419 (95.9) | 333 (94.9) | 86 (100) | 1.000 | 0.051 |
| 3 | 31 (7.1) | 30 (8.5) | 1 (1.2) |  | 406 (92.9) | 321 (91.5) | 85 (98.8) | 0.988 | 0.085 |
| 4 | 50 (11.4) | 49 (14) | 1 (1.2) |  | 387 (88.6) | 302 (86) | 85 (98.8) | 0.988 | 0.140 |
| **5** | 75 (17.2) | 71 (20.2) | 4 (4.7) |  | 362 (82.8) | 280 (79.8) | 82 (95.3) | 0.953 | 0.202 |
| 6 | 105 (24) | 97 (27.6) | 8 (9.3) |  | 332 (76) | 254 (72.4) | 78 (90.7) | 0.907 | 0.276 |
| **7** | 128 (29.3) | 117 (33.3) | 11 (12.8) |  | 309 (70.7) | 234 (66.7) | 75 (87.2) | 0.872 | 0.333 |
| 8 | 147 (33.6) | 135 (38.5) | 12 (14) |  | 290 (66.4) | 216 (61.5) | 74 (86) | 0.860 | 0.385 |
| 9 | 172 (39.4) | 153 (43.6) | 19 (22.1) |  | 265 (60.6) | 198 (56.4) | 67 (77.9) | 0.779 | 0.436 |
| 10 | 184 (42.1) | 165 (47) | 19 (22.1) |  | 253 (57.9) | 186 (53) | 67 (77.9) | 0.779 | 0.470 |
| 11 | 200 (45.8) | 176 (50.1) | 24 (27.9) |  | 237 (54.2) | 175 (49.9) | 62 (72.1) | 0.721 | 0.501 |
| 12 | 219 (50.1) | 193 (55) | 26 (30.2) |  | 218 (49.9) | 158 (45) | 60 (69.8) | 0.698 | 0.550 |
| 13 | 230 (52.6) | 203 (57.8) | 27 (31.4) |  | 207 (47.4) | 148 (42.2) | 59 (68.6) | 0.686 | 0.578 |
| 14 | 242 (55.4) | 211 (60.1) | 31 (36) |  | 195 (44.6) | 140 (39.9) | 55 (64) | 0.640 | 0.601 |
| 15 | 254 (58.1) | 221 (63) | 33 (38.4) |  | 183 (41.9) | 130 (37) | 53 (61.6) | 0.616 | 0.630 |
| 16 | 269 (61.6) | 234 (66.7) | 35 (40.7) |  | 168 (38.4) | 117 (33.3) | 51 (59.3) | 0.593 | 0.667 |
| 17 | 275 (62.9) | 238 (67.8) | 37 (43) |  | 162 (37.1) | 113 (32.2) | 49 (57) | 0.570 | 0.678 |
| 18 | 285 (65.2) | 246 (70.1) | 39 (45.3) |  | 152 (34.8) | 105 (29.9) | 47 (54.7) | 0.547 | 0.701 |
| 19 | 293 (67) | 250 (71.2) | 43 (50) |  | 144 (33) | 101 (28.8) | 43 (50) | 0.500 | 0.712 |
| 20 | 302 (69.1) | 258 (73.5) | 44 (51.2) |  | 135 (30.9) | 93 (26.5) | 42 (48.8) | 0.488 | 0.735 |
| 21 | 318 (72.8) | 268 (76.4) | 50 (58.1) |  | 119 (27.2) | 83 (23.6) | 36 (41.9) | 0.419 | 0.764 |
| 22 | 323 (73.9) | 271 (77.2) | 52 (60.5) |  | 114 (26.1) | 80 (22.8) | 34 (39.5) | 0.395 | 0.772 |
| 23 | 330 (75.5) | 278 (79.2) | 52 (60.5) |  | 107 (24.5) | 73 (20.8) | 34 (39.5) | 0.395 | 0.792 |
| 24 | 341 (78) | 287 (81.8) | 54 (62.8) |  | 96 (22) | 64 (18.2) | 32 (37.2) | 0.372 | 0.818 |
| 25 | 346 (79.2) | 290 (82.6) | 56 (65.1) |  | 91 (20.8) | 61 (17.4) | 30 (34.9) | 0.349 | 0.826 |
| 26 | 351 (80.3) | 293 (83.5) | 58 (67.4) |  | 86 (19.7) | 58 (16.5) | 28 (32.6) | 0.326 | 0.835 |
| 27 | 356 (81.5) | 296 (84.3) | 60 (69.8) |  | 81 (18.5) | 55 (15.7) | 26 (30.2) | 0.302 | 0.843 |
| 28 | 363 (83.1) | 301 (85.8) | 62 (72.1) |  | 74 (16.9) | 50 (14.2) | 24 (27.9) | 0.279 | 0.858 |
| 29 | 368 (84.2) | 305 (86.9) | 63 (73.3) |  | 69 (15.8) | 46 (13.1) | 23 (26.7) | 0.267 | 0.869 |
| 30 | 370 (84.7) | 307 (87.5) | 63 (73.3) |  | 67 (15.3) | 44 (12.5) | 23 (26.7) | 0.267 | 0.875 |

**Table S5** Analyses of the **Memorial Sloan Kettering Cancer Center** model cut-offs used to discriminate between patients with or without lymph node involvement confirmed at extended pelvic lymph node dissection

| Calculated probabil-  ity of LNI % (cut-off) | Number of patients, n (%) | | | | | | | Sensitivity | Specificity |
| --- | --- | --- | --- | --- | --- | --- | --- | --- | --- |
|  | Below the cut-off (ePLND not recommended) | | |  | Equal to or above the cut-off (ePLND recommended) | | |  |  |
|  | Total | Without LNI | With LNI |  | Total | Without LNI | With LNI |  |  |
| 0 | 0 (0) | 0 (0) | 0 (0) |  | 437 (100) | 351 (100) | 86 (100) | 1.000 | 0.000 |
| 1 | 0 (0) | 0 (0) | 0 (0) |  | 437 (100) | 351 (100) | 86 (100) | 1.000 | 0.000 |
| 2 | 7 (1.6) | 7 (2) | 0 (0) |  | 430 (98.4) | 344 (98) | 86 (100) | 1.000 | 0.020 |
| 3 | 21 (4.8) | 21 (6) | 0 (0) |  | 416 (95.2) | 330 (94) | 86 (100) | 1.000 | 0.060 |
| 4 | 28 (6.4) | 26 (7.4) | 2 (2.3) |  | 409 (93.6) | 325 (92.6) | 84 (97.7) | 0.977 | 0.074 |
| **5** | 37 (8.5) | 35 (10) | 2 (2.3) |  | 400 (91.5) | 316 (90) | 84 (97.7) | 0.977 | 0.100 |
| 6 | 42 (9.6) | 39 (11.1) | 3 (3.5) |  | 395 (90.4) | 312 (88.9) | 83 (96.5) | 0.965 | 0.111 |
| **7** | 56 (12.8) | 52 (14.8) | 4 (4.7) |  | 381 (87.2) | 299 (85.2) | 82 (95.3) | 0.953 | 0.148 |
| 8 | 70 (16) | 64 (18.2) | 6 (7) |  | 367 (84) | 287 (81.8) | 80 (93) | 0.930 | 0.182 |
| 9 | 80 (18.3) | 74 (21.1) | 6 (7) |  | 357 (81.7) | 277 (78.9) | 80 (93) | 0.930 | 0.211 |
| 10 | 98 (22.4) | 87 (24.8) | 11 (12.8) |  | 339 (77.6) | 264 (75.2) | 75 (87.2) | 0.872 | 0.248 |
| 11 | 108 (24.7) | 96 (27.4) | 12 (14) |  | 329 (75.3) | 255 (72.6) | 74 (86) | 0.860 | 0.274 |
| 12 | 124 (28.4) | 110 (31.3) | 14 (16.3) |  | 313 (71.6) | 241 (68.7) | 72 (83.7) | 0.837 | 0.313 |
| 13 | 138 (31.6) | 122 (34.8) | 16 (18.6) |  | 299 (68.4) | 229 (65.2) | 70 (81.4) | 0.814 | 0.348 |
| 14 | 147 (33.6) | 130 (37) | 17 (19.8) |  | 290 (66.4) | 221 (63) | 69 (80.2) | 0.802 | 0.370 |
| 15 | 160 (36.6) | 143 (40.7) | 17 (19.8) |  | 277 (63.4) | 208 (59.3) | 69 (80.2) | 0.802 | 0.407 |
| 16 | 174 (39.8) | 156 (44.4) | 18 (20.9) |  | 263 (60.2) | 195 (55.6) | 68 (79.1) | 0.791 | 0.444 |
| 17 | 184 (42.1) | 166 (47.3) | 18 (20.9) |  | 253 (57.9) | 185 (52.7) | 68 (79.1) | 0.791 | 0.473 |
| 18 | 199 (45.5) | 180 (51.3) | 19 (22.1) |  | 238 (54.5) | 171 (48.7) | 67 (77.9) | 0.779 | 0.513 |
| 19 | 213 (48.7) | 190 (54.1) | 23 (26.7) |  | 224 (51.3) | 161 (45.9) | 63 (73.3) | 0.733 | 0.541 |
| 20 | 229 (52.4) | 202 (57.5) | 27 (31.4) |  | 208 (47.6) | 149 (42.5) | 59 (68.6) | 0.686 | 0.575 |
| 21 | 234 (53.5) | 206 (58.7) | 28 (32.6) |  | 203 (46.5) | 145 (41.3) | 58 (67.4) | 0.674 | 0.587 |
| 22 | 240 (54.9) | 211 (60.1) | 29 (33.7) |  | 197 (45.1) | 140 (39.9) | 57 (66.3) | 0.663 | 0.601 |
| 23 | 246 (56.3) | 217 (61.8) | 29 (33.7) |  | 191 (43.7) | 134 (38.2) | 57 (66.3) | 0.663 | 0.618 |
| 24 | 256 (58.6) | 224 (63.8) | 32 (37.2) |  | 181 (41.4) | 127 (36.2) | 54 (62.8) | 0.628 | 0.638 |
| 25 | 263 (60.2) | 229 (65.2) | 34 (39.5) |  | 174 (39.8) | 122 (34.8) | 52 (60.5) | 0.605 | 0.652 |
| 26 | 274 (62.7) | 238 (67.8) | 36 (41.9) |  | 163 (37.3) | 113 (32.2) | 50 (58.1) | 0.581 | 0.678 |
| 27 | 280 (64.1) | 242 (68.9) | 38 (44.2) |  | 157 (35.9) | 109 (31.1) | 48 (55.8) | 0.558 | 0.689 |
| 28 | 289 (66.1) | 251 (71.5) | 38 (44.2) |  | 148 (33.9) | 100 (28.5) | 48 (55.8) | 0.558 | 0.715 |
| 29 | 294 (67.3) | 255 (72.6) | 39 (45.3) |  | 143 (32.7) | 96 (27.4) | 47 (54.7) | 0.547 | 0.726 |
| 30 | 299 (68.4) | 259 (73.8) | 40 (46.5) |  | 138 (31.6) | 92 (26.2) | 46 (53.5) | 0.535 | 0.738 |

**Table S6 Area under the curve of the six models** in the miN0 patient population

| Model | AUC (95% CI) |
| --- | --- |
| Amsterdam-Brisbane-Sydney | 0.70 (0.63 - 0.78) |
| Briganti 2012 | 0.68 (0.60 - 0.76) |
| Memorial Sloan Kettering Cancer Center | 0.72 (0.65 - 0.80) |
| Muehlematter Model 1 | 0.64 (0.57-0.72) |
| Muehlematter Model 2 | 0.61 (0.52 - 0.70) |
| Muehlematter Model 3 | 0.64 (0.56 - 0.71) |

**Fig. S1** **Model calibration plots of predicted probability versus observed probability of lymph node involvement in miN0 subpopulation** for the Muehlematter models 1 to 3, the Amsterdam-Sydney-Brisbane model, the MSKCC Pre-Radical Prostatectomy nomogram with cores, and the Briganti 2012 nomogram. The dashed lines represent a straight fit, the blue line denotes the calibration curve, the ten blue dots denote tenths of the patient cohort (95% CI). The black bars at the top represent the distribution of predicted probabilities


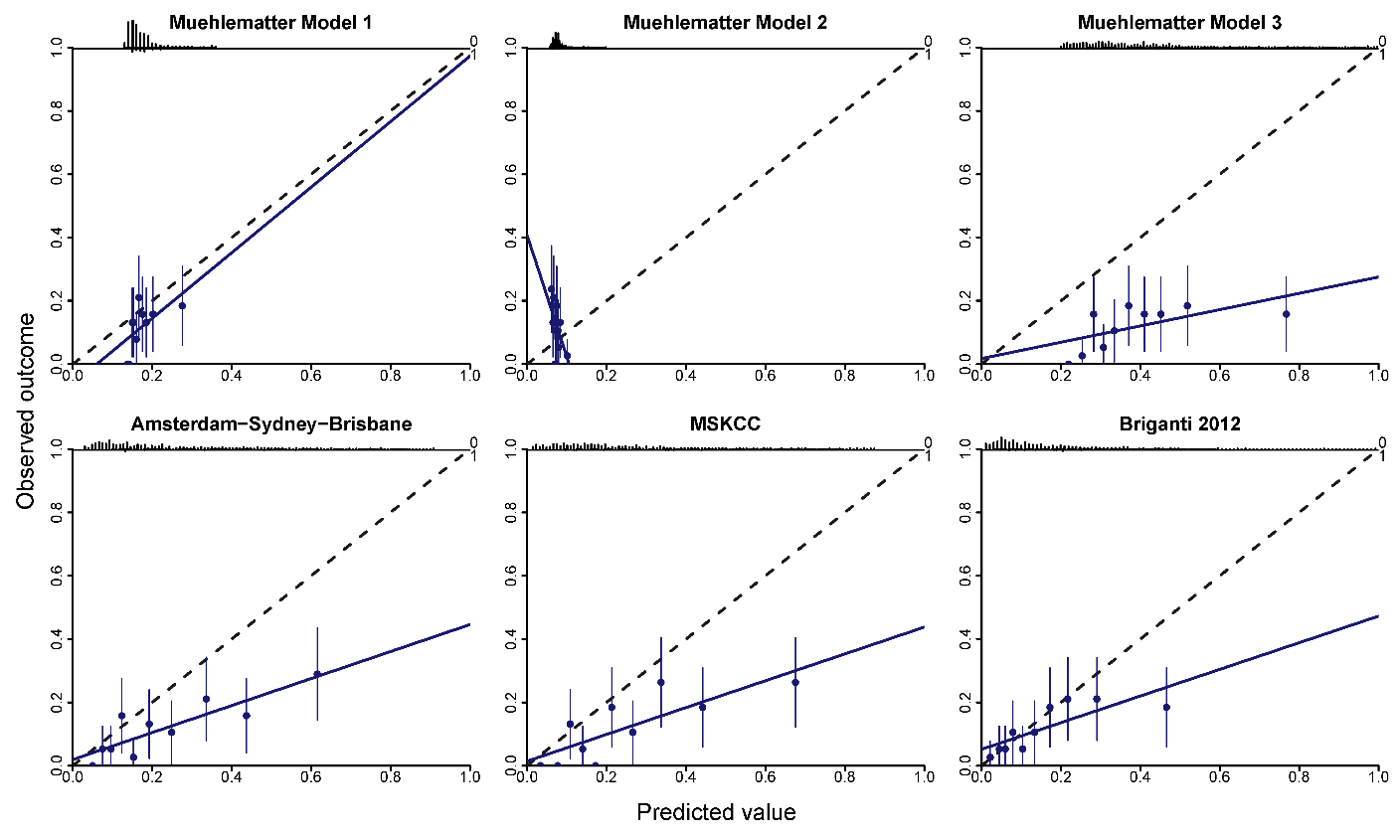


**Fig. S2 Decision curves of all six models in the miN0 population** (Muehlematter Models 1 to 3; Amsterdam-Brisbane-Sydney; MSKCC; Briganti 2012) depicting the net benefit (NB, y-axis) of a model or a strategy (treat-all or treat-none with ePLND) according to a risk threshold (x-axis)


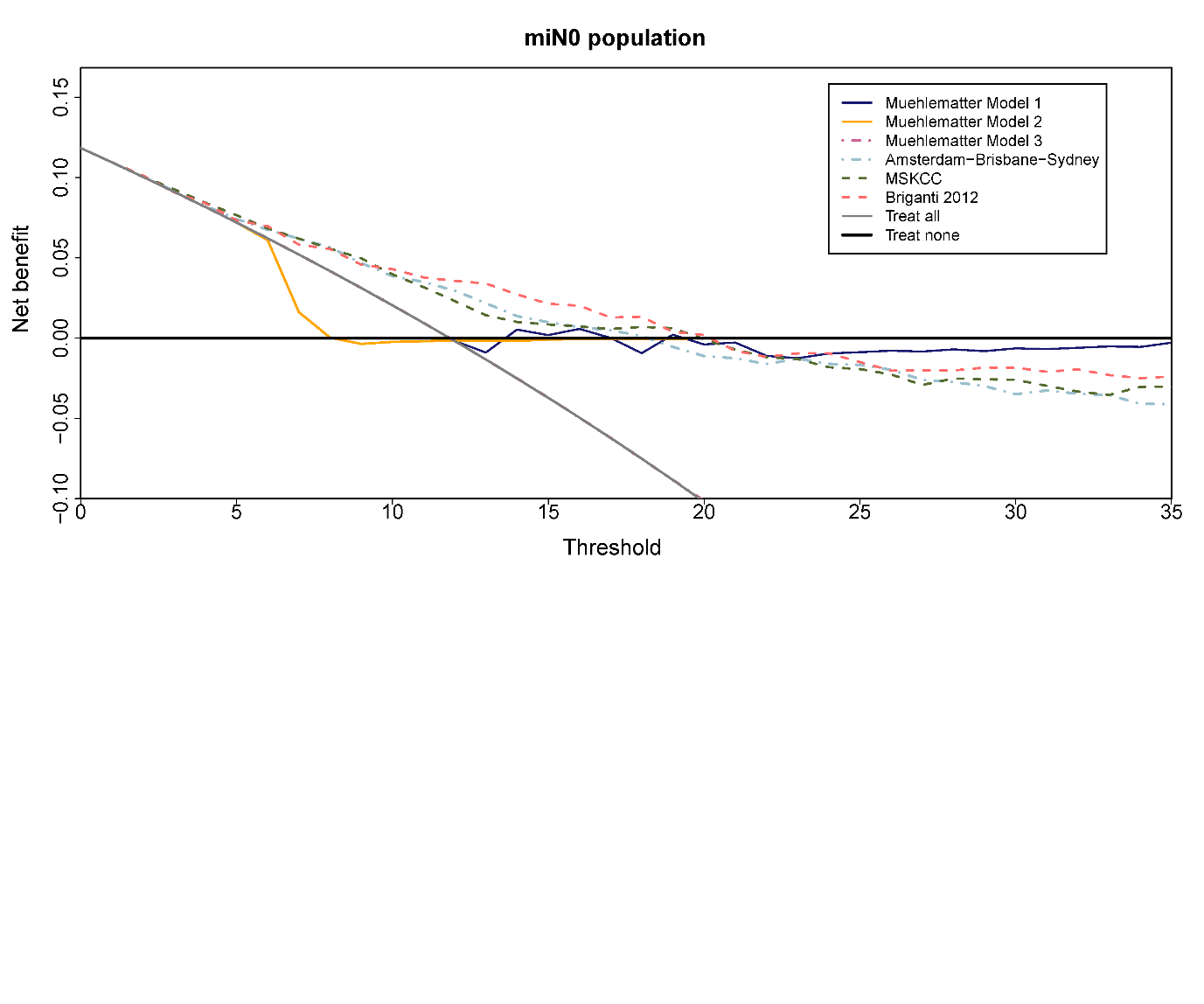


**Table S7 Multivariable logistic regression analysis** containing the variables incorporated in the validated nomograms (right side) (Muehlematter Model 1-3; Amsterdam-Brisbane-Sydney; MSKCC; Briganti 2012) for predicting lymph node invasion in patients treated with radical prostatectomy and extended pelvic lymph node dissection. Left columns show the multivariable logistic regression analysis of the variables that remained after backward elimination using a stepwise selection algorithm guided by Akaike information criterion (AIC)

| Parameter | All variables | | *Final model (after backward elimination)* | |
| --- | --- | --- | --- | --- |
|  | OR (95% CI) | *p-*value | OR (95% CI) | *p-*value |
| Intercept | -4.0311 | | -3.6909 | |
| MR Radiological T stage |  | |  | |
| mrT1-2 | Reference | | Reference | |
| mrT3a | 1.70 (0.87-3.29) | 0.12 | 1.88 (1.00-3.55) | 0.051 |
| MrT3b-4 | 2.49 (1.04-5.95) | 0.040* | 2.90 (1.29-6.55) | 0.010* |
| Biopsy GG according to ISUP |  | |  | |
| 1-2 | Reference | | Reference | |
| 3 | 2.55 (1.07-6.09) | 0.036* | 2.45 (1.03-5.84) | 0.048* |
| 4-5 | 2.55 (1.13-5.77) | 0.024* | 2.60 (1.17-5.77) | 0.043* |
| Percentage positive cores | 2.11 (0.80-5.53) | 0.13 | 2.57 (1.01-6.54) | 0.019* |
| PSMA-PET findings |  | |  | |
| IN0 | Reference | | Reference | |
| IN1 | 20.1 (9.76-41.03) | <0.001*** | 19.87 (9.84-40.13) | <0.001*** |
| PSA | 1.01 (0.98-1.03) | 0.5 | Not included | |
| Age | 1.00 (0.96-1.04) | 0.99 | Not included | |
| PSMAvol | 1.00 (0.98-1.02) | 0.8 | Not included | |
| Clinical stage |  | | Not included | |
| cT1 | Reference | |  |  |
| cT2 | 1.82 (0.94-3.52) | 0.074 |  |  |
| cT3 | 1.84 (0.75-4.51) | 0.18 |  |  |

**Table S8** Diagnostic accuracy measures of PSMA PET/CT for LNI detection among intermediate-risk patients

|  | Complete dataset (n = 174) | | [^68^Ga]Ga-PSMA-11 (n = 95) | | [^18^F]F-PSMA-1007  (n= 77) | |
| --- | --- | --- | --- | --- | --- | --- |
|  | n/N | Result, %  (95% CI) | n/N | Result, %  (95% CI) | n/N | Result, %  (95% CI) |
| Sensitivity | 10/22 | 45.5 (24.4 - 67.8) | 3/9 | 33.3 (7.5 - 70.1) | 7/13 | 53.8 (25.1 - 80.8) |
| Specificity | 145/152 | 95.4 (90.7 - 98.1) | 82/86 | 95.3 (88.5 - 98.7) | 62/64 | 96.9 (89.2 - 99.6) |
| PPV | 10/17 | 58.8 (32.9 - 81.6) | 3/7 | 42.9 (9.9 - 81.6) | 7/9 | 77.8 (40.0 - 97.2) |
| NPV | 145/157 | 92.4 (87.0 - 96.0) | 82/88 | 93.2 (85.7 - 97.5) | 62/68 | 91.2 (81.8 - 96.7) |

**Table S9** Diagnostic accuracy measures of PSMA PET/CT for LNI detection among high-risk patients

|  | Complete dataset (n = 260) | | [^68^Ga]Ga-PSMA-11 (n = 162) | | [^18^F]F-PSMA-1007  (n= 97) | |
| --- | --- | --- | --- | --- | --- | --- |
|  | n/N | Result, %  (95% CI) | n/N | Result, %  (95% CI) | n/N | Result, %  (95% CI) |
| Sensitivity | 31/64 | 48.4 (35.8 - 61.3) | 21/39 | 53.8 (37.2 - 69.9) | 10/25 | 40.0 (21.1 - 61.3) |
| Specificity | 187/196 | 95.4 (91.5 - 97.9) | 116/123 | 94.3 (88.6 - 97.7) | 70/72 | 97.2 (90.3 - 99.7) |
| PPV | 31/40 | 77.5 (61.5 - 89.2) | 21/28 | 75.0 (55.1 - 89.3) | 10/12 | 83.3 (51.6 - 97.9) |
| NPV | 187/220 | 85.0 (79.6 - 89.4) | 116/134 | 86.6 (79.6 - 91.8) | 70/85 | 82.4 (72.6 - 89.8) |

**Table S10** Upgrading and downgrading rates in the overall population. Overall downstaging rate: 154/436 = 35%. Upstaging rate: 64/436 = 15%. 218/436 = 50% correct

| Overall population | | | | | | | |
| --- | --- | --- | --- | --- | --- | --- | --- |
| Biopsy |  | Final histopathology | | | | | |
|  | Grade Group | 1 | 2 | 3 | 4 | 5 | Total |
|  | 1 | 3 (21) | 6 (43) | 4 (29) | 0 (0) | 1 (7) | 14 |
|  | 2 | 2 (2) | 75 (74) | 20 (20) | 3 (3) | 1 (1) | 101 |
|  | 3 | 2 (2) | 41 (33) | 73 (58) | 7 (6) | 3 (2) | 126 |
|  | 4 | 0 (0) | 26 (20) | 49 (39) | 33 (26) | 19 (15) | 127 |
|  | 5 | 0 (0) | 9 (13) | 20 (29) | 5 (7) | 34 (50) | 68 |
|  |  | 7 | 157 | 166 | 48 | 58 | 436 |

**Table S11** Upgrading and downgrading rates in the [^68^Ga]Ga-PSMA-11 subgroup. Overall downstaging rate: 98/258 = 38%. Upstaging rate: 34/258 = 13%. 126/258 = 49% correct

| [^68^Ga]Ga-PSMA-11 | | | | | | | |
| --- | --- | --- | --- | --- | --- | --- | --- |
| Biopsy |  | Final histopathology | | | | | |
|  | Grade Group | 1 | 2 | 3 | 4 | 5 | Total |
|  | 1 | 0 (0) | 3 (60) | 2 (40) | 0 (0) | 0 (0) | 5 |
|  | 2 | 1 (2) | 44 (73) | 13 (22) | 1 (2) | 1 (2) | 60 |
|  | 3 | 1 (1) | 25 (35) | 43 (60) | 3 (4) | 0 (0) | 72 |
|  | 4 | 0 (0) | 21 (27) | 29 (37) | 17 (22) | 11 (14) | 78 |
|  | 5 | 0 (0) | 7 (16) | 12 (28) | 2 (5) | 22 (51) | 43 |
|  |  | 2 | 100 | 99 | 23 | 34 | 258 |

**Table S12** Upgrading and downgrading rates in the [^18^F]F-PSMA-1007 subgroup. Downstaging rate 56/178 = 31%. Upstaging rate 30/178 = 17%. 92/178 = 52% correct

| [^18^F]F-PSMA-1007 | | | | | | | |
| --- | --- | --- | --- | --- | --- | --- | --- |
| Biopsy |  | Final histopathology | | | | | |
|  | Grade Group | 1 | 2 | 3 | 4 | 5 | Total |
|  | 1 | 3 (33) | 3 (33) | 2 (22) | 0 (0) | 1 (11) | 9 |
|  | 2 | 1 (2) | 31 (76) | 7 (17) | 2 (5) | 0 (0) | 41 |
|  | 3 | 1 (2) | 16 (30) | 30 (56) | 4 (7) | 3 (6) | 54 |
|  | 4 | 0 (0) | 5 (10) | 20 (41) | 16 (33) | 8 (16) | 49 |
|  | 5 | 0 (0) | 2 (8) | 8 (32) | 3 (12) | 12 (48) | 25 |
|  |  | 5 | 57 | 67 | 25 | 24 | 178 |

**Table S13 Sensitivity analysis of the AUC** for the Amsterdam-Sydney-Brisbane nomogram (A: counting all cores per prostate lobe if one was ISUP GG ≥2 and B: counting a maximum number of 1 positive core per prostate lobe)

| Tracer | Model | AUC (95% CI) |
| --- | --- | --- |
| Overall population (n = 437) | Amsterdam-Brisbane-Sydney A | 0.81 (0.76 - 0.86) |
|  | Amsterdam-Brisbane-Sydney B | 0.79 (0.74 - 0.85) |
| [^68^Ga]Ga-PSMA-11 (n = 259) | Amsterdam-Brisbane-Sydney A | 0.82 (0.75 - 0.89) |
|  | Amsterdam-Brisbane-Sydney B | 0.82 (0.75 - 0.89) |
| [^18^F]F-PSMA-1007  (n= 178) | Amsterdam-Brisbane-Sydney A | 0.80 (0.72 - 0.88) |
|  | Amsterdam-Brisbane-Sydney B | 0.76 (0.67 - 0.85) |

**Fig. S3** **Sensitivity analysis of model calibration plots** of predicted probability versus observed probability of lymph node involvement for the Amsterdam-Sydney-Brisbane (ABS) nomogram. (a) Calibration plot for ABS-A (counting all cores per prostate lobe if one was ISUP GG ≥2). (b) Calibration plot for ABS-B (counting a maximum number of 1 positive core per prostate lobe with GG ≥2). (c) Calibration plot for both ABS-A (dark-blue) and ABS-B (light-blue).The dashed lines represent a straight fit, the (dark/light-blue line denotes the calibration curve, the ten blue dots denote tenths of the patient cohort (95% CI). The black (a,b) or blue (c) bars on the top denote the distribution of predicted probabilities


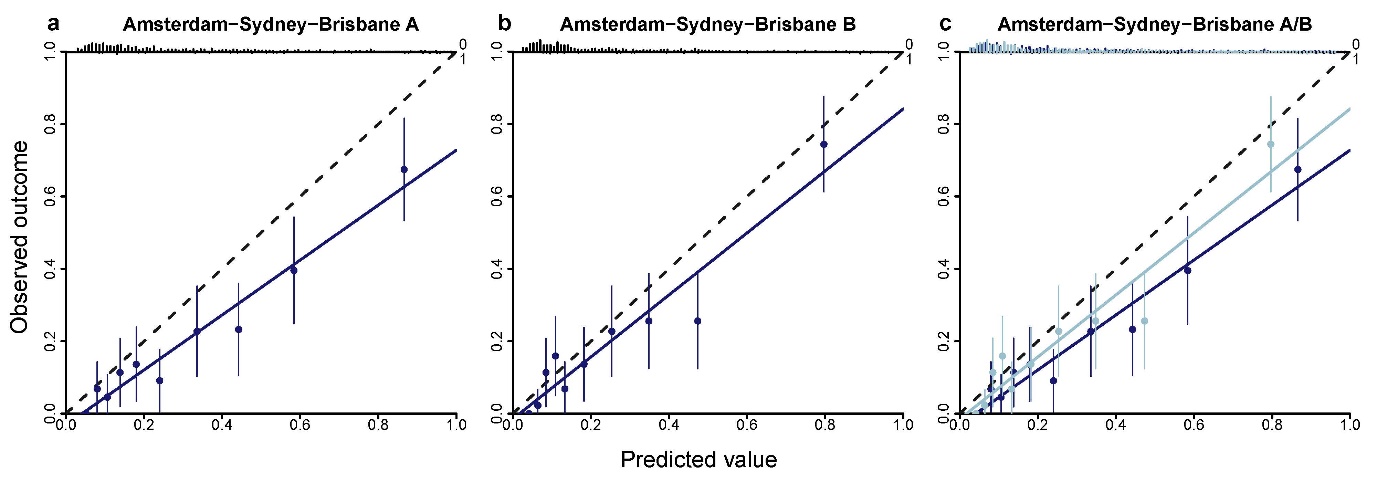


**Fig. S4 Sensitivity analysis of decision curves** for the Amsterdam-Sydney-Brisbane nomogram (A: counting all cores per prostate lobe if one was ISUP GG ≥2 and B: counting a maximum number of 1 positive core per prostate lobe) in the overall population


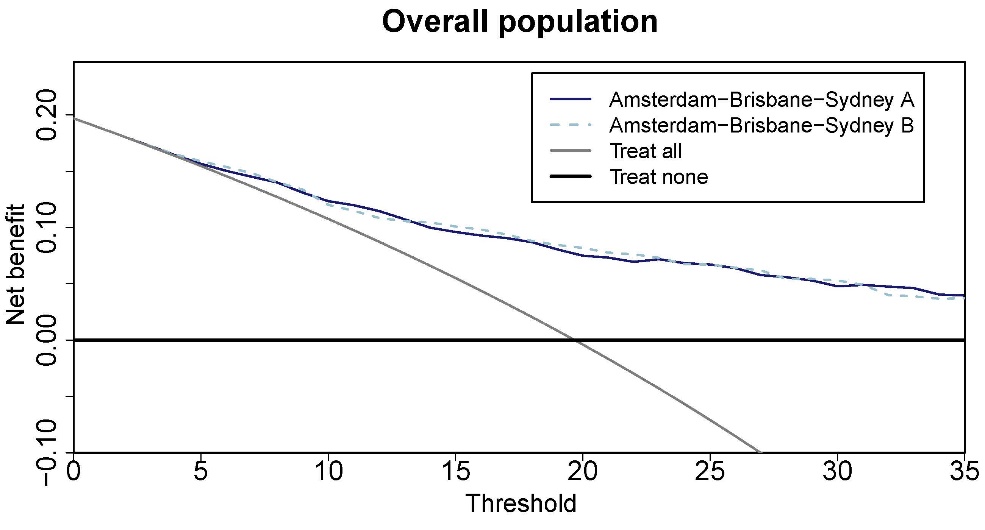


**Table S14** Sensitivity analysis of the Amsterdam-Brisbane-Sydney (ABS-B) cut-offs used to discriminate between patients with or without lymph node involvement confirmed at extended pelvic lymph node dissection

| Calculated probabil-  ity of LNI % (cut-off) | Number of patients, n (%) | | | | | | | Sensitivity | Specificity |
| --- | --- | --- | --- | --- | --- | --- | --- | --- | --- |
|  | Below the cut-off (ePLND not recommended) | | |  | Equal to or above the cut-off (ePLND recommended) | | |  |  |
|  | Total | Without LNI | With LNI |  | Total | Without LNI | With LNI |  |  |
| 0 | 0 (0) | 0 (0) | 0 (0) |  | 437 (100) | 351 (100) | 86 (100) | 1.000 | 0.000 |
| 1 | 0 (0) | 0 (0) | 0 (0) |  | 437 (100) | 351 (100) | 86 (100) | 1.000 | 0.000 |
| 2 | 0 (0) | 0 (0) | 0 (0) |  | 437 (100) | 351 (100) | 86 (100) | 1.000 | 0.000 |
| 3 | 6 (1.4) | 6 (1.7) | 0 (0) |  | 431 (98.6) | 345 (98.3) | 86 (100) | 1.000 | 0.017 |
| 4 | 16 (3.7) | 16 (4.6) | 0 (0) |  | 421 (96.3) | 335 (95.4) | 86 (100) | 1.000 | 0.046 |
| 5 | 35 (8) | 35 (10) | 0 (0) |  | 402 (92) | 316 (90) | 86 (100) | 1.000 | 0.100 |
| 6 | 56 (12.8) | 56 (16) | 0 (0) |  | 381 (87.2) | 295 (84) | 86 (100) | 1.000 | 0.160 |
| 7 | 83 (19) | 82 (23.4) | 1 (1.2) |  | 354 (81) | 269 (76.6) | 85 (98.8) | 0.988 | 0.234 |
| **8** | 104 (23.8) | 101 (28.8) | 3 (3.5) |  | 333 (76.2) | 250 (71.2) | 83 (96.5) | 0.965 | 0.288 |
| 9 | 116 (26.5) | 112 (31.9) | 4 (4.7) |  | 321 (73.5) | 239 (68.1) | 82 (95.3) | 0.953 | 0.319 |
| 10 | 139 (31.8) | 130 (37) | 9 (10.5) |  | 298 (68.2) | 221 (63) | 77 (89.5) | 0.895 | 0.370 |
| **11** | 152 (34.8) | 142 (40.5) | 10 (11.6) |  | 285 (65.2) | 209 (59.5) | 76 (88.4) | 0.884 | 0.405 |
| 12 | 176 (40.3) | 163 (46.4) | 13 (15.1) |  | 261 (59.7) | 188 (53.6) | 73 (84.9) | 0.849 | 0.464 |
| 13 | 192 (43.9) | 178 (50.7) | 14 (16.3) |  | 245 (56.1) | 173 (49.3) | 72 (83.7) | 0.837 | 0.507 |
| 14 | 210 (48.1) | 195 (55.6) | 15 (17.4) |  | 227 (51.9) | 156 (44.4) | 71 (82.6) | 0.826 | 0.556 |
| 15 | 220 (50.3) | 204 (58.1) | 16 (18.6) |  | 217 (49.7) | 147 (41.9) | 70 (81.4) | 0.814 | 0.581 |
| 16 | 231 (52.9) | 214 (61) | 17 (19.8) |  | 206 (47.1) | 137 (39) | 69 (80.2) | 0.802 | 0.610 |
| 17 | 237 (54.2) | 219 (62.4) | 18 (20.9) |  | 200 (45.8) | 132 (37.6) | 68 (79.1) | 0.791 | 0.624 |
| 18 | 240 (54.9) | 221 (63) | 19 (22.1) |  | 197 (45.1) | 130 (37) | 67 (77.9) | 0.779 | 0.630 |
| 19 | 247 (56.5) | 227 (64.7) | 20 (23.3) |  | 190 (43.5) | 124 (35.3) | 66 (76.7) | 0.767 | 0.647 |
| 20 | 250 (57.2) | 230 (65.5) | 20 (23.3) |  | 187 (42.8) | 121 (34.5) | 66 (76.7) | 0.767 | 0.655 |
| 21 | 260 (59.5) | 238 (67.8) | 22 (25.6) |  | 177 (40.5) | 113 (32.2) | 64 (74.4) | 0.744 | 0.678 |
| 22 | 264 (60.4) | 242 (68.9) | 22 (25.6) |  | 173 (39.6) | 109 (31.1) | 64 (74.4) | 0.744 | 0.689 |
| 23 | 270 (61.8) | 247 (70.4) | 23 (26.7) |  | 167 (38.2) | 104 (29.6) | 63 (73.3) | 0.733 | 0.704 |
| 24 | 276 (63.2) | 251 (71.5) | 25 (29.1) |  | 161 (36.8) | 100 (28.5) | 61 (70.9) | 0.709 | 0.715 |
| 25 | 285 (65.2) | 259 (73.8) | 26 (30.2) |  | 152 (34.8) | 92 (26.2) | 60 (69.8) | 0.698 | 0.738 |
| 26 | 290 (66.4) | 263 (74.9) | 27 (31.4) |  | 147 (33.6) | 88 (25.1) | 59 (68.6) | 0.686 | 0.749 |
| 27 | 299 (68.4) | 270 (76.9) | 29 (33.7) |  | 138 (31.6) | 81 (23.1) | 57 (66.3) | 0.663 | 0.769 |
| 28 | 305 (69.8) | 273 (77.8) | 32 (37.2) |  | 132 (30.2) | 78 (22.2) | 54 (62.8) | 0.628 | 0.778 |
| 29 | 309 (70.7) | 277 (78.9) | 32 (37.2) |  | 128 (29.3) | 74 (21.1) | 54 (62.8) | 0.628 | 0.789 |
| 30 | 311 (71.2) | 279 (79.5) | 32 (37.2) |  | 126 (28.8) | 72 (20.5) | 54 (62.8) | 0.628 | 0.795 |
